# Cellular senescence promotes macrophage-to-myofibroblast transition in chronic ischemic renal disease

**DOI:** 10.1038/s41419-025-07666-1

**Published:** 2025-05-10

**Authors:** Yu Zhao, Xiang-Yang Zhu, Wenqi Ma, Ying Zhang, Fei Yuan, Seo Rin Kim, Hui Tang, Kyra Jordan, Amir Lerman, Tamara Tchkonia, James L. Kirkland, Lilach O. Lerman

**Affiliations:** 1 Institute of Nephrology, Zhong Da Hospital, Southeast University, School of Medicine, Nanjing, Jiangsu PR China; 2 Division of Nephrology and Hypertension, Mayo Clinic, Rochester, MN USA; 3 Department of Cardiovascular Medicine, Ruijin Hospital, Shanghai Jiao Tong University School of Medicine, Shanghai, PR China; 4 Department of Urology, National Children’s Medical Center, Shanghai Children’s Medical Center, School of Medicine, Shanghai Jiao Tong University, Shanghai, PR China; 5 Department of Cardiovascular Diseases, Mayo Clinic, Rochester, MN USA; 6 Department of Physiology and Biomedical Engineering, Mayo Clinic, Rochester, MN USA; 7 Division of General Internal Medicine, Mayo Clinic, Rochester, MN USA

**Keywords:** Senescence, Renal artery stenosis

## Abstract

Cellular senescence participates in the pathophysiology of post-stenotic kidney damage, but how it regulates tissue remodeling is incompletely understood. Macrophage-myofibroblast transition (MMT) contributes to the development of tissue fibrosis. We hypothesized that cellular senescence contributes to MMT and renal fibrosis in mice with renal artery stenosis (RAS). *INK-ATTAC* mice expressing *p16*^*INK-4a*^ and green fluorescent protein in senescent cells were assigned to control or unilateral RAS, untreated or treated with AP20187 (an apoptosis inducer in *p16*^*INK-4a*^-expressing cells) for 4 weeks. Renal perfusion was studied in vivo using micro-MRI, and kidney morphology, senescence, and MMT ex vivo. Cellular senescence was induced in human renal proximal tubular epithelial cells (HRPTEpiC) in vitro, and interferon-induced transmembrane protein-3 (IFITM3), a cellular senescence vector, was silenced (siRNA) or over-expressed (plasmid). HRPTEpiC were then co-incubated with macrophages with silenced integrin-3 (ITGB3), a regulator of mesenchymal transitions. CD68/*p16*^*INK-4a*^/α-SMA co-expression and senescence markers were studied. Murine RAS kidneys showed increased expression of *p16*^*INK-4a*^ and MMT markers (F4/80, α-SMA) vs. controls, which decreased after AP20187, as did renal fibrosis and plasma creatinine, whereas renal perfusion increased. IFITM3 and ITGB3 expression were upregulated in senescent HRPTEpiC or co-cultured macrophages, respectively. MMT markers and TGF-β/Smad3 expression also rose in these macrophages and decreased after IFITM3 or ITGB3 silencing. *p16*^*INK-4a*^-expressing macrophages may regulate interstitial fibrosis in RAS via MMT. This process is associated with elevated expression of ITGB3 and TGF-β/Smad3 pathway activation through neighboring senescent cell-derived IFITM3. These findings may implicate MMT as a therapeutic target in ischemic kidneys.

## Introduction

Renal artery stenosis (RAS) is a major cause of renovascular hypertension and may lead to end-stage kidney disease. Kidney damage distal to the stenosis is characterized by inflammation, microvascular loss, and interstitial fibrosis. Experimental studies have shown that macrophage infiltration can exacerbate kidney damage, leading to fibrosis and microvascular damage in the stenotic kidney. Congruently, we found a correlation between the progression of macrophages and macrophage-derived cytokines and the severity of the disease in human post-stenotic kidneys [1, 2]. In order to establish effective therapeutic strategies, it is crucial to identify the mechanisms underlying the macrophage-induced fibrosis in RAS.

Recent studies in mice with unilateral ureteral obstruction and patients with renal allograft injury have shown that macrophages are capable of transitioning into myofibroblasts as identified by co-expression of macrophage markers (F4/80 in mice or CD68 in humans) and α-smooth muscle actin (a-SMA) [3, 4]. The process of macrophage-to-myofibroblast transition (MMT) is driven by transforming growth factor-β (TGF-β)/Smad3 signaling [5]. MMT may serve as a key checkpoint for the progression of chronic inflammation into pathogenic fibrosis [6] in chronic kidney diseases, but its contribution to renal fibrosis in RAS remains unknown.

Cellular senescence is characterized by cell cycle arrest, development of a proinflammatory senescence-associated secretory phenotype (SASP), macromolecular damage, and metabolic disorders [6]. Senescent cells not only have anti-apoptotic and pro-survival defenses but frequently express the pro-inflammatory SASP, which consolidates senescence and adversely influences adjacent cells, contributing to pathogenesis in aging and chronic diseases [7]. Recently, interferon-induced transmembrane protein-3 (IFITM3) [8], a membrane protein associated with inflamed phenotypes and cancer, has been identified as a key vector of paracrine transmission of senescence from senescent cells to nearby parenchymal cells. In the kidney, senescent cells aggravate senescence in adjacent cells in acute [8] and chronic ischemic injury [9]. However, whether and how they communicate with macrophages to modulate chronic ischemic renal damage remains unclear.

In macrophages, surface expression of integrin-β3 (ITGB3) may mediate polarization toward a pro-fibrotic M2 phenotype [10], regulate the microenvironment, and facilitate epithelial-to-mesenchymal and endothelial-to-mesenchymal transitions [11] in concert with TGF-β. Conceivably, ITGB3 expression might also regulate MMT in macrophages, but its role is incompletely understood.

Therefore, we hypothesized that cellular senescence contributes to MMT and renal fibrosis in mice with chronic RAS, and that senescent parenchymal cell IFITM3 and macrophage ITGB3 contribute to their inter-cellular communication in this setting.

## Methods

**Fig. 1:**
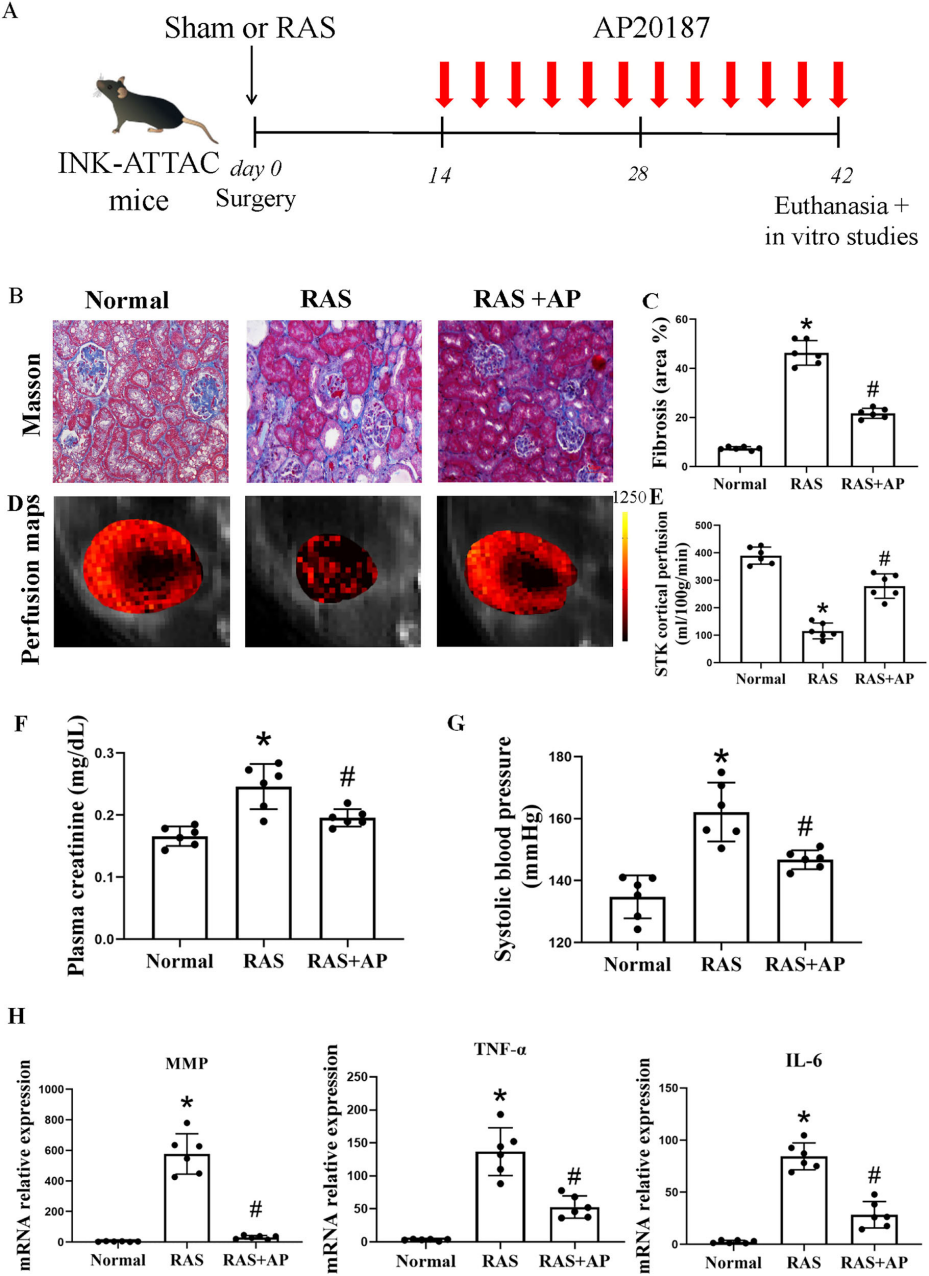
Chronic ischemia induces fibrosis in the stenotic kidney (STK), while AP20187 (AP) improves renal function and perfusion. **A** Schematic representation of the experimental protocol. **B**, **D** Masson’s Trichrome staining revealed increased fibrosis in the STK of renal artery stenosis (RAS) mice, which was significantly attenuated by AP treatment. Scale bar: 50 µm. **C** Renal perfusion maps generated by arterial spin-labeling MRI (mL/100 g/min; brighter red indicates higher perfusion) demonstrated reduced STK perfusion in RAS mice, which was restored in RAS + AP20187 (**E**). Plasma creatinine levels, elevated in RAS mice, were reduced following AP treatment (**F**). Additionally, renal gene expression of proinflammatory and profibrotic factors was blunted in RAS + AP20187 (**G**). The expression of senescence-associated secretory phenotype (SASP) genes, including IL-6, MMP3, and TNF-α, was markedly elevated in RAS compared to normal kidneys but significantly decreased with AP treatment (**H**). Data are mean ± SD (*n* = 6/group). *P < 0.05 vs. Normal; ^#^*P* < 0.05 vs. RAS.

All animal studies were approved by the Mayo Clinic Institutional Animal Care and Use Committee. We used in these experiments mice heterozygous for the *INK-ATTAC* transgenes (a transgenic mouse model that expresses FKBP-Casp8 and GFP under the control of a minimal *p16*^*INK-4a*^ promoter fragment transcriptionally active in senescent cells) on a female *C57BL/6* background [12–14]. For RAS induction, 10-week-old mice were anesthetized and surgically implanted with a 0.15 mm diameter plastic arterial cuff around a right renal artery, whereas sham surgery included the same process but without cuff placement [9]. Successful RAS surgery was confirmed by subsequent right-to-left volume or weight ratio of less than 0.9, and no mice were excluded in this study. Two weeks later, RAS mice were randomly assigned to 3.3 mg/kg AP20187 (B/B homodimerizer, Clontech, Mountain View, CA) or vehicle injections 3 times a week [15] (*n* = 6 each). Our previous studies confirmed that a sample size of *n* = 6 allows detection of statistically significant changes in the RAS kidney, including shrinkage and hypoperfusion [9, 16]. AP20187 activates caspase-8 and selectively induces apoptosis of cells highly expressing *p16*^*INK-4a*^, with little effect on normal cells. In subsets of RAS mice, a low dose of liposomal clodronate 100 μl (FormuMax Scientific CA) was injected intraperitoneally every 4 days for 4 weeks, as we have shown before [17]. Mice were maintained under a 12 h light and 12 h dark cycle at 24 °C with free access to food and water in a pathogen-free facility. Two weeks later, stenotic-kidney cortical perfusion was measured using micro-magnetic resonance imaging (MRI) with arterial spinning labeling as per previous protocols [9]. Urine and blood samples were taken for measurement of biochemical parameters, the mice were euthanized, and the kidneys collected for histological and molecular assays (Fig. 1A). Plasma creatinine level was assayed using DetectX® kit (Arbor Assays, Ann Arbor, MI).

### Ex-vivo studies

Kidney sections were stained with Masson’s trichrome staining and examined by light microscopy (×40) in a blinded manner. Twenty randomly selected non-overlapping slides from each kidney were analyzed using Image-J software (NIH, USA).

Total RNA was isolated from frozen mouse kidneys by mirVana PARIS total RNA isolation, and its concentration was measured by a NanoDrop Spectrophotometer (both from Thermo-Fisher Scientific, Waltham, MA). Relative quantitative PCR for SASP factors was performed using Taqman assays, containing 10 ng of cDNA products, IL-6 (mm00446190), MMP-3 (mm00440295), TNF-α (mm00443258), and GAPDH (mm99999915) as internal control. Analysis was done on Applied Biosystems ViiA7 Real-Time PCR systems. Fold-changes of each target gene in the experimental relative to control groups were calculated using the 2^−ΔΔCT^ method.

For immunofluorescence, the kidney tissue was stained for F4/80 (ab6640, Abcam, USA) and α-SMA (ab7817, Abcam). After staining the nuclei with DAPI, F4/80 and α-SMA immunostaining, and *p16*^*INK-4a*^ (by GFP) was visualized with a fluorescence microscope (×400).

Senescence-associated β-galactosidase (SA-β-gal) staining was performed using a Kit (Cell Signaling, Boston, MA), as described [18] with eosin counterstaining. The SA–β–gal–positive area was determined in ten randomly chosen fields per section at ×20 magnification, assessed for the development of blue color, and expressed as an average to the total field area [19].

### In vitro studies

Firstly, human renal proximal tubular epithelial cells (HRPTEpiC) (ScienCell, Carlsbad, CA) [20, 21] were grown in an epithelial cell medium (ScienCell) containing epithelial cell growth supplement (ScienCell), in a humidified atmosphere of 95% air and 5% CO_2_ at 37 °C. Cells were plated (3 × 10^5^ cells/well) in 6-well plates and incubated for 3 days. We compared various approaches to induce senescence in HRPTEpiC, including H_2_O_2_ (60 µM, 1 h; 100 µM, 24 h), TNF-α (20 ng/mL, 72 h; 10 ng/mL, 7 days), and TNF-α (10 ng/mL) + TGF-β (5 ng/mL), 3 days [22, 23]. HRPTEpiC mRNA expression of *p16*^*INK-4a*^*, p21*^*Cip1/Waf1*^, p53, IL-6, TNF-α, PAI-1, and MCP-1 was measured to ascertain development of senescence.

Secondly, macrophages were isolated by the gradient centrifugation method from peripheral blood obtained from healthy human volunteers. Cells were then cultured for 7 days with RPMI 1640 media supplemented with 10% fetal bovine serum and 50 ng/ml M-CSF (R&D, MN, USA). All procedures were approved by Zhongda Hospital Affiliated to Southeast University Clinical Research Ethics Committee, and informed consent was obtained from all subjects.

To manipulate the expression of genes implicated in inter-cellular communication, lipid complexes containing IFITM3 siRNA or plasmid (both Gene-Pharma) were formed by incubating them with Lipofectamine 2000, and used to pre-treat some senescent HRPTEpiC, whereas macrophages were untreated or pre-treated with ITGB3 siRNA or plasmid (both Gene-Pharma). In a transwell co-culture system (Costar polycarbonate filters, 0.4 μm pores), untreated or IFITM3 pre-treated senescent HRPTEpiCs were randomly seeded to the upper chamber, and untreated or ITGB3 pre-treated macrophages were added to the lower chamber for 72 h.

Cellular SA-β-gal was stained using the SA-β-Galactosidase Staining Kit (Cell Signaling). Briefly, cells were washed, fixed, and incubated overnight with the staining solution. Cells were then examined for the development of blue color, photographed at ×20 magnification (Zeiss microscopy, Germany), and analyzed by Image-J.

For assessment of senescence by immunofluorescence, macrophages fixed with 4% formaldehyde were incubated with anti-CD68 (ab95564, Abcam), *p16*^*INK-4a*^ (10883-1-AP, Protein tech), α-SMA (48938S, Cell Signaling), or Ki67 (AF7617, R&D Systems) and then a secondary antibody. After staining the nuclei with DAPI, the cells were analyzed by Image-J.

The senescence and fibrosis markers were further tested by standard western blotting protocols [24] using specific antibodies against *p16*^*INK-4a*^ (10883-1-AP, Protein tech), *p21*^*Cip1/Waf1*^ (00094204, Protein tech), TGF-β (ab215715, Abcam), Smad3 (ab40854, Abcam), α-SMA (ab40854, Abcam), collagen-I (ab260043, Abcam), IFITM3 (SAB1404822, Sigma-Aldrich), ITGB3 (SAB4300361, Sigma-Aldrich), p53 (32532S, Cell-Signaling),p-p53.S15 (AF1043-SP, Bio-Techne), γ-H2AX (sc-517336, Santa Cruze), p-Smad 2/3 (Ser 423/425) (sc-11769, Santa Cruze). β-actin or GAPDH was used as loading control. Protein expression was averaged in each group. Additionally, gene expression of the senescence and SASP markers *p16*^*INK-4a*^ (mm00494449), *p21*^*Cip1/Waf1*^ (mm00432448), p53 (mm01731290), TNF-α (mm00443258), IL-1α (mm00439620), IL-6 (mm00446190), MCP-1 (mm00441242), and PAI-1 (mm00436753) was also measured by RT-PCR. β-actin was used as loading control. IFITM3 (abx250358, abbexa) in HRPTEpiC supernatant was tested by ELISA.

### Statistical analysis

Data were analyzed using GraphPad Prism-5 (GraphPad Software, San Diego, CA, USA), and results are expressed as mean ± standard deviation for normally distributed variables, and median (range) for non-Gaussian distributed data. Comparisons within groups were performed using the paired Student’s *t*-test, and among groups using ANOVA and unpaired *t*-test with Bonferroni correction. A statistical difference was considered significant for *p* ≤ 0.05.

## Results

### Identification of MMT in *INK-ATTAC* RAS mice

We used *INK-ATTAC* mice, which express *p16*^*INK-4a*^ promoter-driven green fluorescent protein exclusively in senescent cells, to investigate whether a subset of a-SMA^+^ myofibroblasts in the fibrosing kidney derives from the *p16*^*INK-4a*^-positive macrophages. When renal fibrosis was developed, stenotic-kidney cortical perfusion was decreased, plasma creatinine was elevated, and systolic blood pressure (SBP) was increased after RAS induction in *INK-ATTAC* mice (Fig. 1). Furthermore, mRNA expression of the proinflammatory and profibrotic SASP genes, IL-6, MMP3, and TNF-α, was markedly elevated compared to Normal (Fig. 1).

**Fig. 2:**
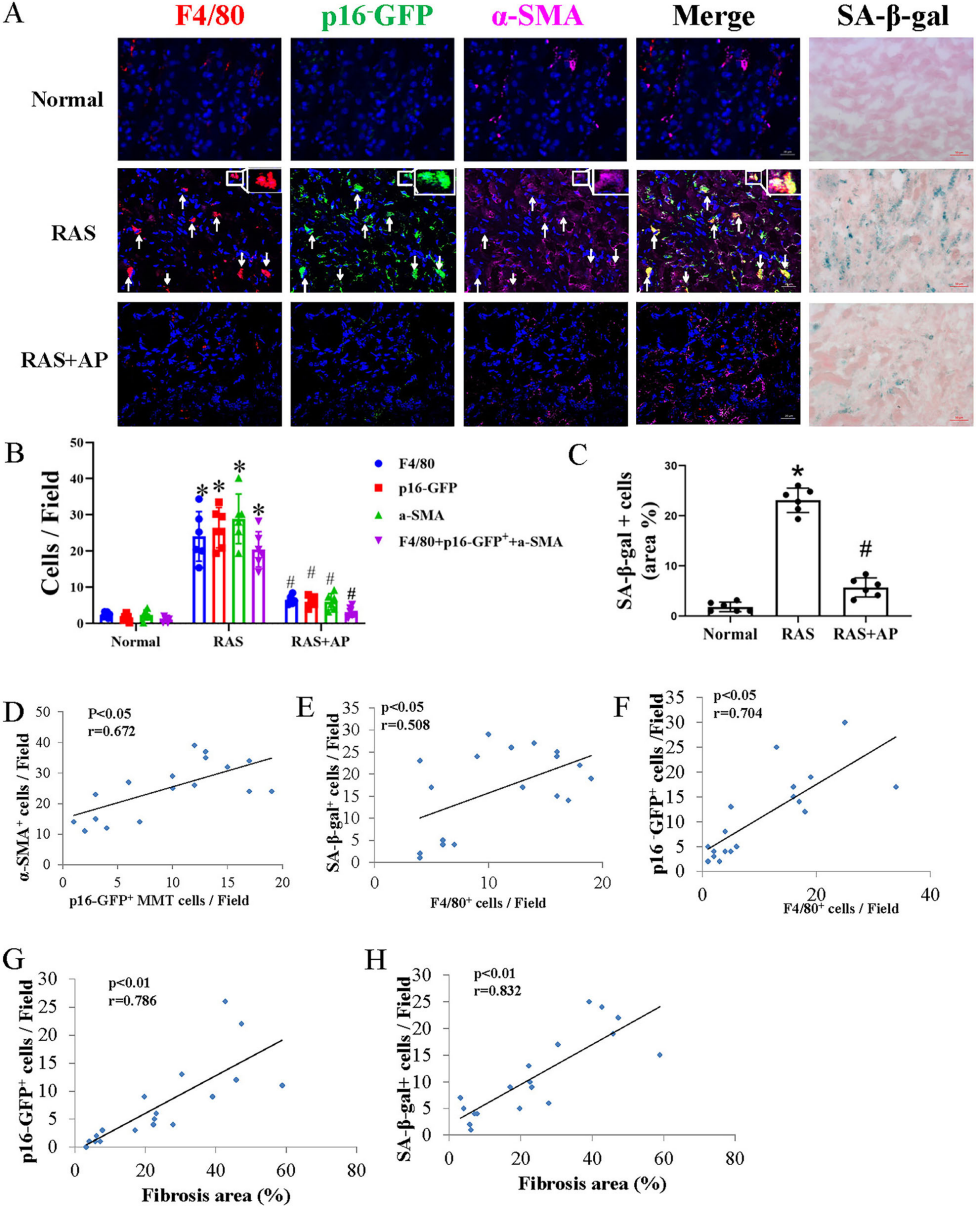
Senescent cell clearance decreases MMT. Triple-immunofluorescence images (**A**) and quantification (**B**) identified MMT cells in RAS kidneys that co-express macrophage (F4/80, red), senescence activation (p16-GFP, green), and myofibroblast (α-SMA, pink) markers, compared to controls (Normal). These MMT cell populations were significantly reduced in RAS kidneys treated with the *p16*^*INK-4a+*^ cell apoptosis inducer, AP20187 (RAS + AP20187). A positive correlation was observed between MMT cell populations and the number of α-SMA^+^ cells (**D**). The senescence marker SA-β-gal activity, which was elevated in RAS compared to normal kidneys, showed a significant reduction following AP20187 treatment (**A**, **C**). Scale bar: 20 µm (immunofluorescence), 50 µm (SA-β-gal). **E**, **F** The numbers of F4/80^+^ macrophages correlated positively with the numbers of *p16*^*INK-4a+*^ and SA-β-gal^+^ cells in both normal and RAS kidneys. **G**, **H** Similarly, renal fibrosis correlated strongly with the numbers of *p16*^*INK-4a+*^ and SA-β-gal^+^ cells in the mouse kidneys. Data are mean ± SD (*n* = 6 per group). *P < 0.05 vs. Normal; #*P* < 0.05 vs. RAS. p16-GFP: *p16*^*INK-4a+*^ labeled by green fluorescent protein (GFP).

In RAS mice with interstitial fibrosis, F4/80^+^*p16*^*INK-4a*+^a-SMA^+^ cells were detected in the stenotic kidney using confocal microscopy, indicating that *p16*^*INK-4a+*^ macrophages were the source of the MMT process. These F4/80^+^*p16*^*INK-4a*+^a-SMA^+^ MMT cells accounted for approximately 50% of the total a-SMA^+^ myofibroblast population, and F4/80^+^*p16*^*INK-4a+*^ cells accounted for nearly 80% of the total F4/80^+^ cells. Normal mice showed fewer F4/80^+^*p16*^*INK-4a* +^a-SMA^+^ MMT cells compared with RAS mice (Fig. 2). Ultimately, the number of *p16*^*INK-4a+*^ MMT cells correlated with a-SMA^+^ myofibroblasts (*p* < 0.05, *r* = 0.672, Fig. 2).

To clarify the role of macrophage in MMT, we treated RAS mice with clodronate to ablate macrophages and found their number decreased by 45%. We have shown before that this regimen diminishes but does not fully eradicate macrophages [17]. With the reduction of macrophages, *p16*^*INK-4a*^ and a-SMA were, respectively, reduced by 20 and 28%, suggesting the notion that approximately 50% macrophages were involved in MMT (Fig. S1).

### Senescent cell clearance decreases MMT

To investigate whether *p16*^*INK-4a+*^ cells directly drive MMT and whether targeting senescent cells can reduce fibrosis in RAS-affected kidneys, we induced senescent cell clearance in mice using AP20187. Treatment with AP20187 decreased plasma creatinine levels, improved cortical perfusion in stenotic kidneys, and strongly trended toward reducing tubulointerstitial fibrosis and pro-inflammatory marker expression.

The number of MMT cells in RAS kidneys was significantly reduced following AP20187 treatment, indicating that senescent cell clearance mitigates the enhanced *p16*^*INK-4a+*^-associated MMT. Similarly, the senescence marker SA-β-gal, which was elevated in RAS compared to normal kidneys, showed a significant reduction in RAS + AP20187-treated mice (Fig. 2).

Furthermore, we observed a direct correlation between senescence markers and F4/80^+^ macrophages, as well as between senescence markers and the degree of tubulointerstitial fibrosis (Fig. 2). These findings suggest that senescence is closely linked to the severity of fibrosis in RAS kidneys and that clearing senescent cells can alleviate fibrosis and inflammation in this context.

### Senescent cells induce MMT in-vitro

**Fig. 3:**
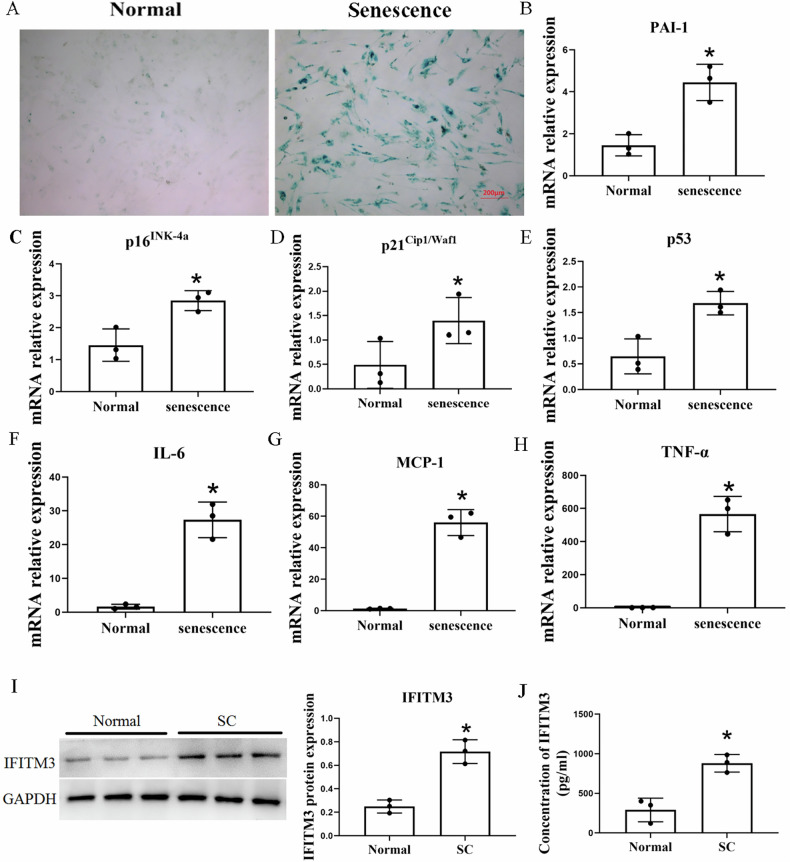
TNF-α and TGF-β induce senescence in HRPTEpiC. SA-β-gal staining (**A**) and the mRNA expression of senescence markers (PAI-1, *p16*^*INK-4a*^, *p21*^*Cip1/Waf1*^, p53, IL-6, MCP-1, TNF-α) (**B**–**H**) were significantly increased in human renal proximal tubular epithelial cells (HRPTEpiC) following TNF-α and TGF-β treatment. Similarly, the protein levels of IFITM3 were elevated under the same conditions (**I**, **J**). Data are mean ± SD (*n* = 3/group). *P < 0.05 vs. Normal. PAI-1: plasminogen activator inhibitor-1. Scale bar: 200 µm (**A**).

Senescent cells and macrophages were co-cultured in a transwell system to verify the direct effect of senescent cells on macrophages and identify the mechanisms underlying the MMT. First, we determined that the combination of TNF-α (10 ng/mL) and TGF-β (5 ng/mL) for 72 h was the most effective among the different methods tested for inducing HRPTEpiC senescence (Fig. S2). Then, HRPTEpiC were treated with TNF-α and TGF-β for 72 h, which increased the number of SA-β-gal^+^ cells and the expression of the senescence markers *p16*^*INK-4a*^, *p21*^*Cip1/Waf1*^, p53, IL-6, TNF-α, PAI-1, and MCP-1 by RT-PCR (Fig. 3).

**Fig. 4:**
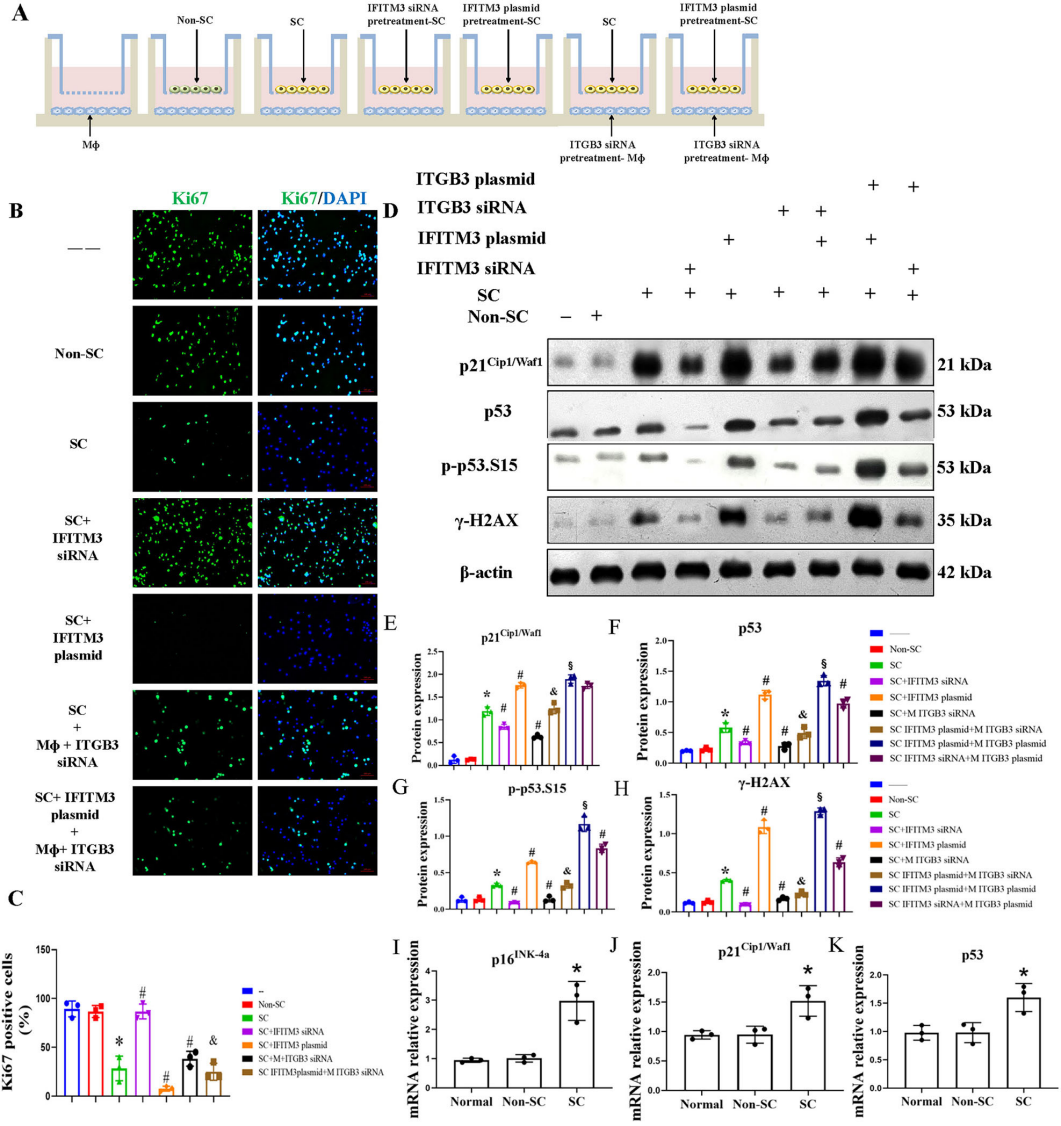
Senescent cells (SC) induce senescence in macrophages. **A** Schematic of the in vitro experimental protocol. **B**, **C**: Immunofluorescence staining of Ki67 and quantitative analysis of Ki67^+^ cells. The number of Ki67^+^ cells decreased after co-culture with senescent human renal proximal tubular epithelial cells (HRPTEpiC) (senescent cells, SC). Scale bar: 100 µm (**B**). The data are mean ± SD (n = 3/group). **D**–**H**: The effects of IFITM-3 and ITGB-3 on macrophage senescence. IFITM3 and ITGB3 were manipulated in SC HRPTEpiC and macrophages, respectively. Macrophages were subsequently collected for Western blot analysis of senescence markers *p21*^*Cip1/Waf1*^, p53, p-p53.S15, and γ-H2AX. Silencing ITGB3 or IFITM3 individually using siRNA reduced the expression of these senescence markers. However, this blunting effect was mitigated when IFITM3 was overexpressed in HRPTEpiC, indicating that IFITM3 plays a critical role in maintaining senescence signaling despite ITGB3 knockdown. Data are mean ± SD (*n* = 3/group). *P < 0.05 vs. Normal control (NC) and Non-SC groups; ^#^*P* < 0.05 vs. SC group; and *P* < 0.05 vs. SC + IFITM3 over-expressing groups. **I**–**K**: Relative mRNA expression of the senescence markers *p16*^*INK-4a*^*, p21*^*Cip1/Waf1*^, and p53 increased in macrophages co-incubated with senescent cells. β-actin was used as loading control. Data are mean ± SD (*n* = 3/group). *P < 0.05 vs. Normal.

**Fig. 5:**
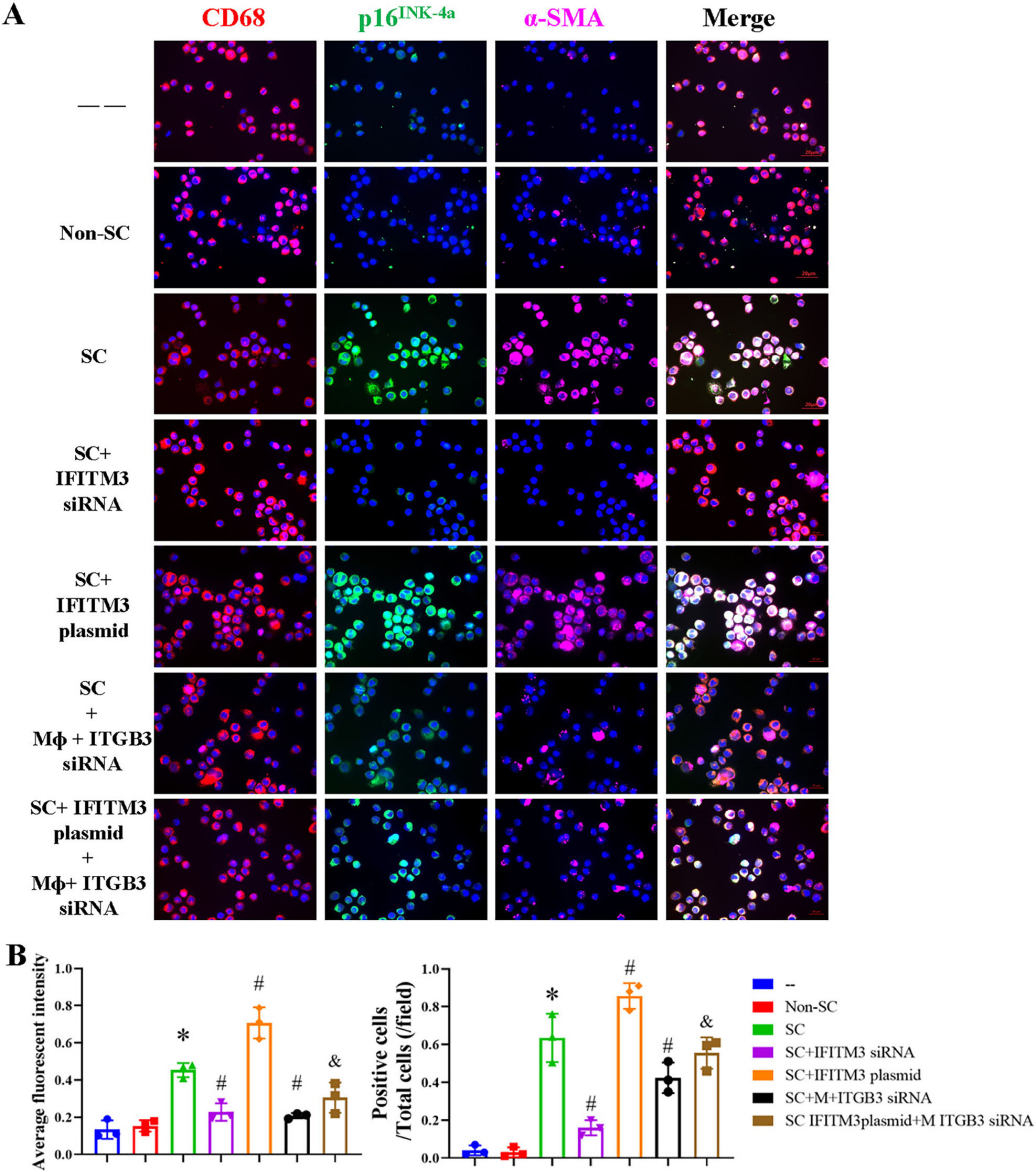
IFITM3 and ITGB3 regulate MMT in macrophages. **A** Triple immunofluorescence analysis identified MMT cells co-expressing macrophage (CD68, red), senescence activation (*p16*^*INK-4a*^, green), and myofibroblast (α-SMA, pink) markers under different experimental conditions. Scale bar: 20 µm. **B** Semi-quantitative analysis of average fluorescence intensity for CD68, *p16*^*INK-4a*^, and α-SMA-positive cells demonstrated that silencing ITGB3 or IFITM3 using siRNA significantly reduced the colocalization of these markers. In contrast, treatment with an IFITM3 plasmid increased the immunofluorescent colocalization of CD68, *p16*^*INK-4a*^, and α-SMA, suggesting a role for IFITM3 in promoting MMT marker expression. Data are mean ± SD (*n* = 3/group). *P < 0.05 vs. Normal control (NC) and Non-SC groups; #*P* < 0.05 vs. SC group; and *P* < 0.05 vs. SC + IFITM3 over-expressing groups.

Next, we plated senescence cells in the upper and macrophages in the lower chamber of the transwell. To determine development of cellular arrest in macrophages. We stained them with Ki67. We observed that the overall number of Ki67^+^ cells decreased after co-culture with senescent cells, indicating that senescent cells led to decreased proliferation ability of macrophage. Importantly, the protein levels of *p21*^*Cip1/Waf1*^, p53, p-p53 (serine 15), and γ-H2AX in macrophages increased after co-culture with senescent HRPTEpiC, supporting macrophages underwent senescence. The mRNA expression of senescence markers showed a similar trend in that *p16*^*Ink-4a*^, *p21*^*Cip1/Waf1*^, and *p53* increased in macrophages after senescent cell treatment compared with untreated macrophages (Fig. 4). CD68, p16^INK-4a^, and α-SMA, observed by immuno-labeling and confocal microscopy imaging, showed increased colocalization in macrophages after senescence cell treatment compared with untreated macrophages (Fig. 5). These data suggest that senescent cells directly induce MMT and senescence marker expression in macrophages.

In order to determine whether specifically MMT macrophages are senescent, we stained the cells with triple immunofluorescence. The data show that MMT macrophages co-incubated with senescent cells exhibit decreased proliferation ability, consistent with senescence, although the proportion of senescent cells is higher than in vivo (Fig. S3). Therefore, in vitro experiments confirmed that macrophages with senescent cells induced MMT develop senescent phenotypes (Fig. S4).

### IFITM3 in senescent cells mediates their communication with macrophages

IFITM3 expression was significantly increased in both senescent cells and supernatant (Fig. 3). To investigate its involvement in the interaction between senescent cells and macrophages, siRNA targeting IFITM3 was transfected into senescent cells. A non-target control (NTC) siRNA was used to account for any nonspecific effects of the transfection reagents. Because the inhibition ratios of IFITM3 siRNA-1, 2, and 3 were 76.8%, 39.2%, and 21.6%, we selected IFITM3 siRNA-1 for the subsequent siRNA intervention. NTC siRNA had no significant effect on IFITM3 expression (Fig. S5).

To investigate whether inhibition of IFITM3 expression prevents MMT, we then pre-treated HRPTEpiC with IFITM3 siRNA and used transwell inserts to introduce a physical barrier between the cells. We plated non-senescent cells, senescent cells, and IFITM3 siRNA pre-treated senescent cells in the upper chamber and macrophages in the lower chamber. The effect of IFITM3 siRNA on senescence-induced MMT was studied by Western blot (Fig. 4) and immunofluorescence (Fig. 5) and showed a lower expression of *α-*SMA, collagen-I, *p16*^*INK-4a*^, *p21*^*Cip1/Waf1*^, and p53 and fall in immunofluorescence colocalization of CD68, *p16* ^*INK-4a*^, and α-SMA. These data suggest that IFITM3 siRNA prevents senescent cell-induced MMT.

### The role of ITGB3 in the IFITM3-regulated MMT

In senescence cells, ITGB3, as a member of the integrin family, has been extensively studied. Both ITGB3 α-β heterodimers, αvβ3 and αIIβ3, serve as receptors for vitronectin [25]. It is significantly upregulated and may be involved in activating MMT [26, 27]. To further clarify its effect on MMT, siRNA targeting ITGB3 was transfected into macrophages. The inhibition ratios of ITGB3 siRNA-1, 2, and 3 were 0.7%, 24.3%, and 69.8%. Therefore, we used ITGB3 siRNA-3 for the subsequent final intervention (Fig. S5), and its effects on MMT were observed by Western blot (Fig. 4) and immunofluorescence (Fig. 5). We observed decreased immunofluorescence colocalization of CD68, *p16*^*INK-4a*^, and α-SMA and lower expression of collagen-I and *p21*^*Cip1/Waf1*^. Therefore, ITGB3 siRNA prevents senescent cell-induced MMT.

**Fig. 6:**
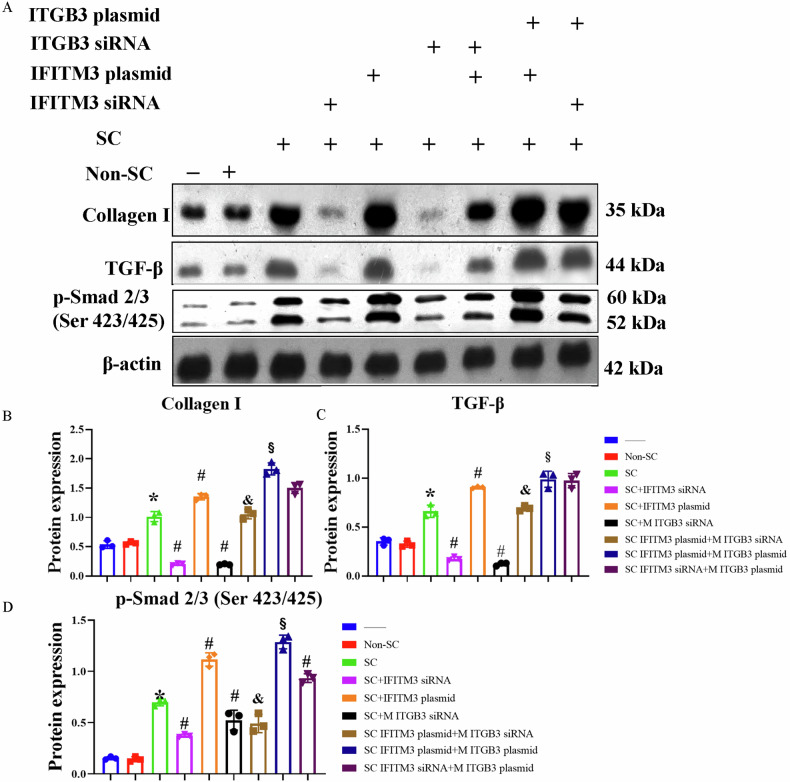
The effects of IFITM-3 and ITGB-3 on MMT in macrophages. **A** IFITM3 and ITGB3 were manipulated in senescent human renal proximal tubular epithelial cells (SC) and macrophages, respectively. Macrophages were subsequently collected for Western blot analysis to assess markers of mesenchymal transition (MMT) like Collagen-I, and pathway-related proteins (p-Smad2/3 and TGF-β). Knockdown of ITGB3 or IFITM3 using siRNA individually reduced the expression of Collagen I (**B**), TGF-β (**C**), and p-Smad2/3 (**D**). However, the blunting effects of siRNA treatment were attenuated when IFITM3 and ITGB3 were overexpressed, highlighting their roles in MMT and pathway activation in macrophages. Data are mean ± SD (*n* = 3/group). *P < 0.05 vs. Normal control (NC) and Non-SC groups; ^#^*P* < 0.05 vs. SC group; and ^&^*P* < 0.05 vs. SC + IFITM3 over-expressing groups; ^§^*p* < 0.05 vs. SC IFITM3 plasmid+M ITGB3 siRNA.

To clarify further the relationship between IFITM3 and ITGB3, on IFITM3 plasmid was transfected into senescent cells with overexpression efficiency (160.6%) determined by Western blot (Fig. S5). Senescent cells treated with the IFITM3 overexpression plasmid were then co-cultured with the ITGB3 siRNA-stimulated macrophages. In contrast to ITGB3 siRNA treatment alone, IFITM3 plasmid treatment increased immunofluorescent colocalization of CD68, *p16*^*INK-4a*^, and α-SMA (Fig. 5) and expression of *p21*^*Cip1/Waf1*^, p53, γ-H2AX (Fig. 4) and Collagen-I (Fig. 6). NTC siRNA used as control in our experiments had no significant inhibiting effects on any of protein targeted in our study (Figs. S5–S6).

### Macrophage-myofibroblast transition pathways

To further elucidate the possible mechanisms of MMT, TGF-β and Smad3 [3, 28] macrophage expression were evaluated. Their expression as well as activation (i.e., p-Smad2/3 [Ser 423/425] expression) increased after co-culture with senescent HRPTEpiC and decreased after IFITM3-siRNA or ITGB3-siRNA treatment. However, these blunting effects on TGF-β/Smad3 expression were attenuated when IFITM3 was over-expressed in HRPTEpiC (Figs. 4–6). To investigate the link between ITGB3 and TGF-β, ITGB3 was overexpressed, and IFITM3 was silenced. We found that Collagen-I, *p21*^*Cip1/Waf1*^, and TGF-β expression were significantly upregulated in SC + IFITM3 plasmid+Mɸ + ITGB3 plasmid group compared with SC + IFITM3 plasmid+Mɸ + ITGB3 siRNA group (Fig. S5). These data suggest that the IFITM3-ITGB3-regulated MMT is associated with upregulation of TGF-β and Smad3.

## Discussion

This study shows that murine RAS induces development of kidney MMT and fibrosis, which decreases after eliminating *p16*^*INK-4a*^-positive cells, suggesting that they were in part secondary to cellular senescence. The crosstalk between senescent epithelial cells and macrophages might be partly mediated by their expression of IFITM3 and ITGB3, respectively, and involves the activation of pro-fibrotic TGF-β/Smad3. These findings imply a key role for senescence-associated MMT in ischemic kidney remodeling and suggest MMT as a therapeutic target.

RAS is the major cause of secondary hypertension and may lead to kidney ischemia and eventually end-stage kidney disease [29]. The mechanisms that contribute to injury in the ischemic kidney include tissue inflammation, microvascular loss, and elevated oxidative stress, resulting in renal fibrosis and dysfunction that play a fundamental role in the development and progression of RAS [30]. Therefore, elucidating the mechanism of inflammation and renal interstitial fibrosis in RAS is of key scientific importance.

Myofibroblasts are important contributors to renal fibrosis. Recent studies provide evidence that macrophages recruited from the bone marrow can transition directly into myofibroblasts within the injured kidney via MMT [3], which may serve as a key checkpoint for the progression of chronic inflammation into pathogenic fibrosis. In 1994, Richard and colleagues defined a new type of fibrocyte, derived from circulating cells, that has fibroblast properties, infiltrates subcutaneously implanted wounds, and participates in tissue repair [31]. Subsequent studies have demonstrated that monocyte/macrophages from peripheral blood can differentiate into fibroblasts [32–34]. Huiyao et al. proposed that bone marrow-derived macrophages undergoing MMT within the fibrotic kidney were a major source of collagen-producing fibroblasts in the fibrosing kidney, accounting for over 60% of α-SMA^+^ myofibroblasts in murine ureteral obstruction [35, 36]. Further studies confirmed that MMT cells account for ~50% of the myofibroblast population, and their numbers correlate with allograft function and the severity of interstitial fibrosis in patients with chronic renal allograft injury [4]. Consistent with these reports, in our study, MMT cells accounted for ~50% of the post-stenotic kidney myofibroblasts. Our results implicate MMT as a key process leading to fibrosis in ischemic renal tissue. However, the mechanism regulating MMT has not been fully elucidated.

Senescence is a cellular phenotype featured by a stable cell-cycle arrest in which an inflammatory response is mediated through the SASP. This is an important hallmark of aging, predominantly mediated by cell cycle regulators p53, *p21*^*Cip1/Waf1*^, and *p16*^*INK-4a*^. In particular, *p16*^*INK-4a*^ regulates cell senescence and can increase the risk of aging-related diseases, including disorders associated with kidney dysfunction [37, 38]. Senescent cells can, in turn, exert noxious effects on neighboring and distant cells, partly disseminated through IFITM3. A variety of insults can transform renal proximal tubular epithelial cells into a senescent phenotype. A biopsy series of patients with different types of renal diseases demonstrated increased *p21*^*Cip1/Waf1*^ and *p16*^*INK-4a*^ protein expression confined to the tubular epithelium and interstitial nuclei [39]. Our previous study identified upregulation of *p16*^*INK-4a*^ gene expression in renal epithelial cells and macrophages of *INK-ATTAC* transgenic mice [9]. Macrophage abundance also correlates with senescent cell burden in adipose tissue of obese mice [12]. The current study extends our previous observations and demonstrates links among renal cellular senescence (SA-β-gal), interstitial fibrosis, α-SMA^+^ cells, *p16*^*INK-4a*+^ cells, and macrophages.

We found that *p16*^*INK-4a*^-positive macrophages account for approximately 80% of macrophages in ischemic kidneys. Yet, *p16*^*INK-4a*^ and SA-β-gal are also upregulated in macrophages during their polarization to an M2-phenotype [40]. Furthermore, activated macrophages in atherosclerotic lesions resemble senescent cells with lipid accumulation, SA-β-gal positivity, and a persistent DNA damage response [41]. Hence, a senescence-like phenotype in macrophages might constitute a physiological activation state adopted in response to challenge, and their *p16*^*INK4a*^-positivity in the ischemic kidney might be at least in part attributable to activated macrophages. Nonetheless, a large fraction also acquired α-SMA-positivity, suggesting that those activated macrophages undergo MMT. As an index of cellular senescence in these cells, Ki67 staining showed that many of the *p16*^*INK-4a*^-positive macrophages were non-proliferating and thus likely senescent both in vivo and in vitro. Possibly, *p16*^*INK-4a+*^ macrophages include both senescent and activated macrophages in vivo, both of which are capable of MMT transdifferentiation.

To explore the effect of cellular senescence on MMT, first, we cleared *p16*^*INK-4a+*^ cells from RAS kidneys using AP20187, which significantly improved renal function, blunted fibrosis, and reduced MMT cell number. Yet, this could have conceivably been achieved by elimination of both senescent parenchymal cells and activated macrophages from RAS kidneys. Thus, to test whether tubular cells' senescence might directly contribute to MMT progression, we co-cultured macrophages with senescent HRPTEpiC in vitro. We found that the expression of the senescence markers *p16*^*INK-4a*^, *p21*^*Cip1/Waf1*^, and p53 all increased in these macrophages, which might indicate that some of these were in fact senescent. This was accompanied by a rise in the number of MMT cells (CD68^+^/α-SMA^+^/*p16*^*INK-4a+*^). These results support the notion that cellular senescence, either intrinsic (macrophage) or extrinsic (HRPTEpiC), may be involved in MMT regulation.

Senescence can be transmitted to healthy cells in a paracrine fashion [42, 43]. Studies have shown that senescent cells have transmitted senescence to nearby cells through IFITM3. For example, Fang found that senescent macrophages aggravate senescence and calcification of vascular smooth muscle cells via IFITM3 [44]. Hur et al. reported that IFITM3 expression increases with age and contributes to elevated γ-secretase activity and Aβ production in Alzheimer’s disease [45], and Wu et al. implicated the cGAS-STING-IFITM3 axis in Aβ-induced neuroinflammation in microglia [46]. Yet, the mechanism by which IFITM3 mediates secondary senescence and the mode and mechanism of communication between senescent cells and macrophages remained still unclear. Some proposed mechanisms included the interferon response, H2O2 production, or both contribute to secondary senescence in macrophages. Li et al. successfully induced secondary senescence in macrophages by exposure to H_2_O_2_ [47]. Interferon has also been reported to induce senescence in cancer cells, melanocytes, endothelial cells, and mesenchymal stem cells [48–50], although its ability to induce macrophage senescence remains to be determined.

We explored the hypothesis that IFITM3 regulates MMT through by ITGB3, which in turn activates via TGF-β/Smad signaling. Indeed, our data showed that IFITM3 expression was upregulated in both senescent HRPTEpiC and macrophages co-cultured with them. Moreover, when senescent HRPTEpiC IFITM3 expression was silenced, MMT was blunted. Interestingly, IFITM3 overexpression by an IFITM3-plasmid amplified some MMT markers (protein expression) with a modest impact on colocalization, suggesting that pathophysiological upregulation of IFITM3 expression in senescent HRPTEpiC is close to maximal. Nevertheless, the IFITM3-plasmid negated the effects of macrophage ITGB3 silencing on MMT, implicating it in regulating MMT.

ITGB3 mediates M2 macrophage polarization [10] and facilitates transitions of both epithelial and endothelial cells towards mesenchymal phenotypes [11]. Hence, we hypothesized that macrophage ITGB3 might regulate MMT. ITGB3 protein may promote both cellular senescence and MMT by regulating the TGF-β/Smad signaling pathway [27], which in turn regulates the transition of bone marrow-derived macrophages into myofibroblasts during tissue fibrosis [5]. Our data show that the expression of ITGB3, TGF-β, and Smad3 significantly rises in macrophages co-cultured with senescent HRPTEpiC, associated with MMT. We also found that MMT markers and TGF-β signaling are attenuated by both ITGB3-siRNA and IFITM3-siRNA, although IFITM3-silencing appeared slightly more effective. Pertinently, the attenuating effect of ITGB3-siRNA on MMT was blunted after IFITM3 overexpression. These results suggest that senescent-cell IFITM3 regulates MMT, which may be at least in part mediated by macrophage ITGB3.

## Limitations

Our studies are limited by the uncertain suitability of *p16*^*INK-4a*^ and SA-β-gal as senescence markers in macrophages, and by the potential AP20187-induced elimination of both senescent parenchymal cells and activated macrophages from RAS kidneys of *INK-ATTAC* transgenic mice. Clearly, the senescent phenotype of activated macrophages and interplay among truly senescent cells and senescent-like macrophages warrant additional studies [51]. Nevertheless, our in vitro studies complimented our in vivo studies to show a direct effect of senescent tubular epithelial cells on macrophage activation, MMT, and pro-fibrogenic mechanisms.

## Conclusions

**Fig. 7:**
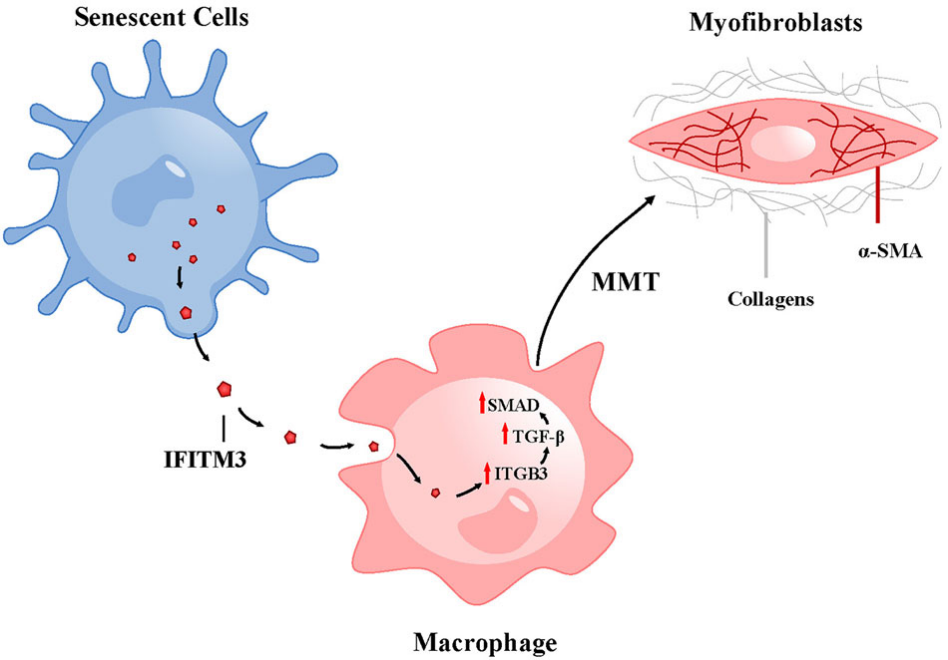
Schematic diagram of senescent cell induction of Macrophage-Myofibroblast Transition (MMT), which may result in tissue fibrosis. We identified a critical role for macrophages in giving rise to myofibroblasts, contributing to interstitial fibrosis in RAS through mesenchymal transition (MMT). This process appears to be mediated, at least in part, by senescent cell-derived IFITM3, which promotes ITGB3 expression and activates the TGF-β/Smad3 signaling pathway.

As presented schematically in Fig. 7, we identify *p16*^*INK-4a+*^ macrophages as an important source for myofibroblasts through the process of MMT, which contributes to interstitial fibrosis in RAS. This process was associated with and may be at least partly mediated by increased expression of macrophage ITGB3 and activation of the TGF-β/Smad3 pathway secondary to senescent cell-derived IFITM3. Future studies are needed to confirm the activation of these processes in injured human kidneys and develop targeted therapeutic interventions.

## Supplementary information

Original Data

Supplemental figures and legends

Western Blot Gels

Reproducability checklist

## Data Availability

The data underlying this article, the study protocol, and the statistical analysis plan will be shared upon reasonable request to the corresponding author.
