# Supplementary material for: Cellular senescence promotes macrophage-to-myofibroblast transition in chronic ischemic renal disease
Source: Cell Death Dis. 2025 May 10;16(1):372. doi: 10.1038/s41419-025-07666-1 (PMC12065848; doi:10.1038/s41419-025-07666-1)
Supplement: Supplementary file 2 — Supplemental figures and legends [file 41419_2025_7666_MOESM2_ESM.pdf]

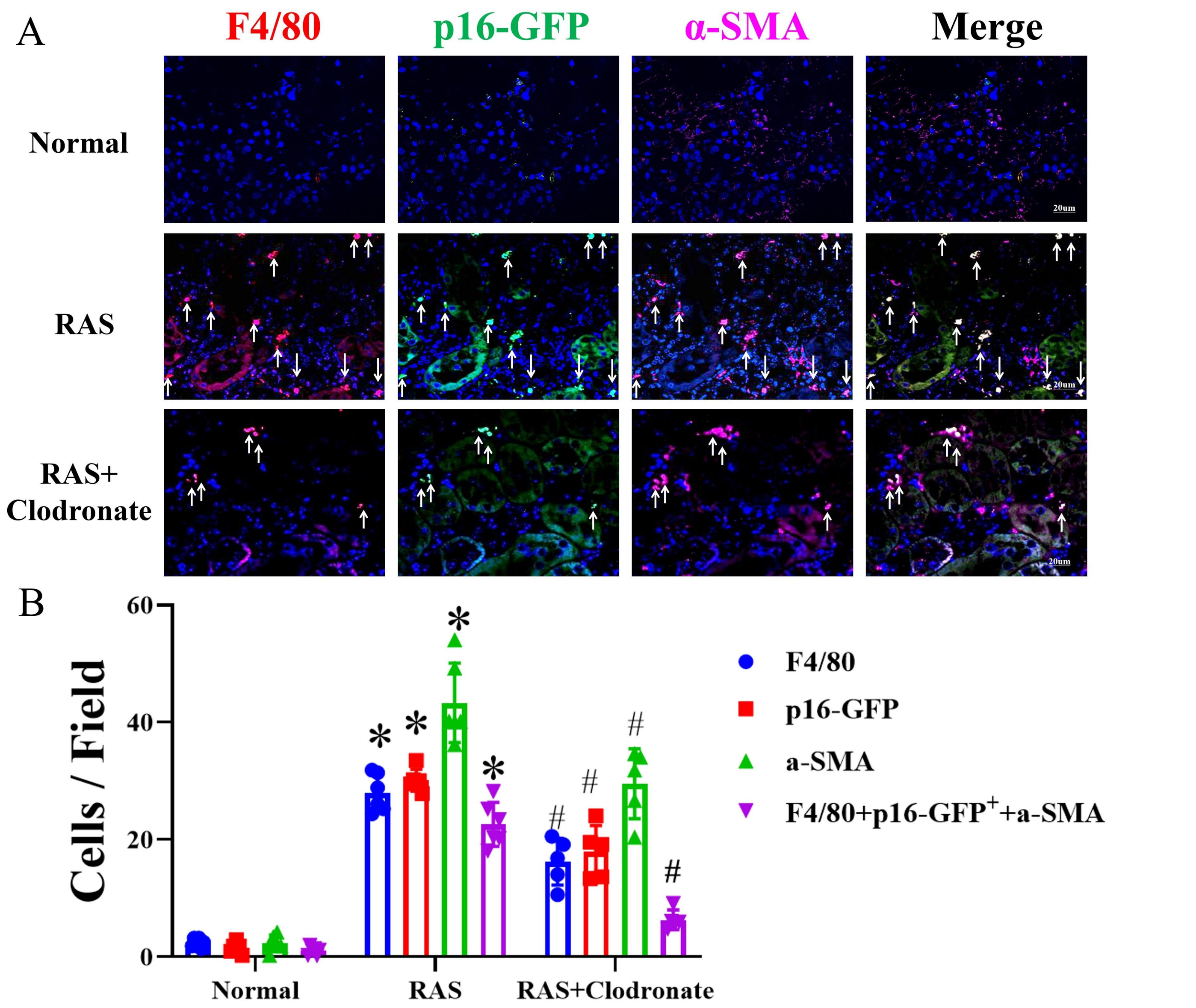
A
F4/80
p16-GFP
α-SMA
Merge
Normal
RAS
RAS+
Clodronate
20um
B
Cells / Field
60
40
20
0
Normal
RAS
RAS+Clodronate
F4/80
p16-GFP
a-SMA
F4/80+p16-GFP+a-SMA
*
#

Fig.S1

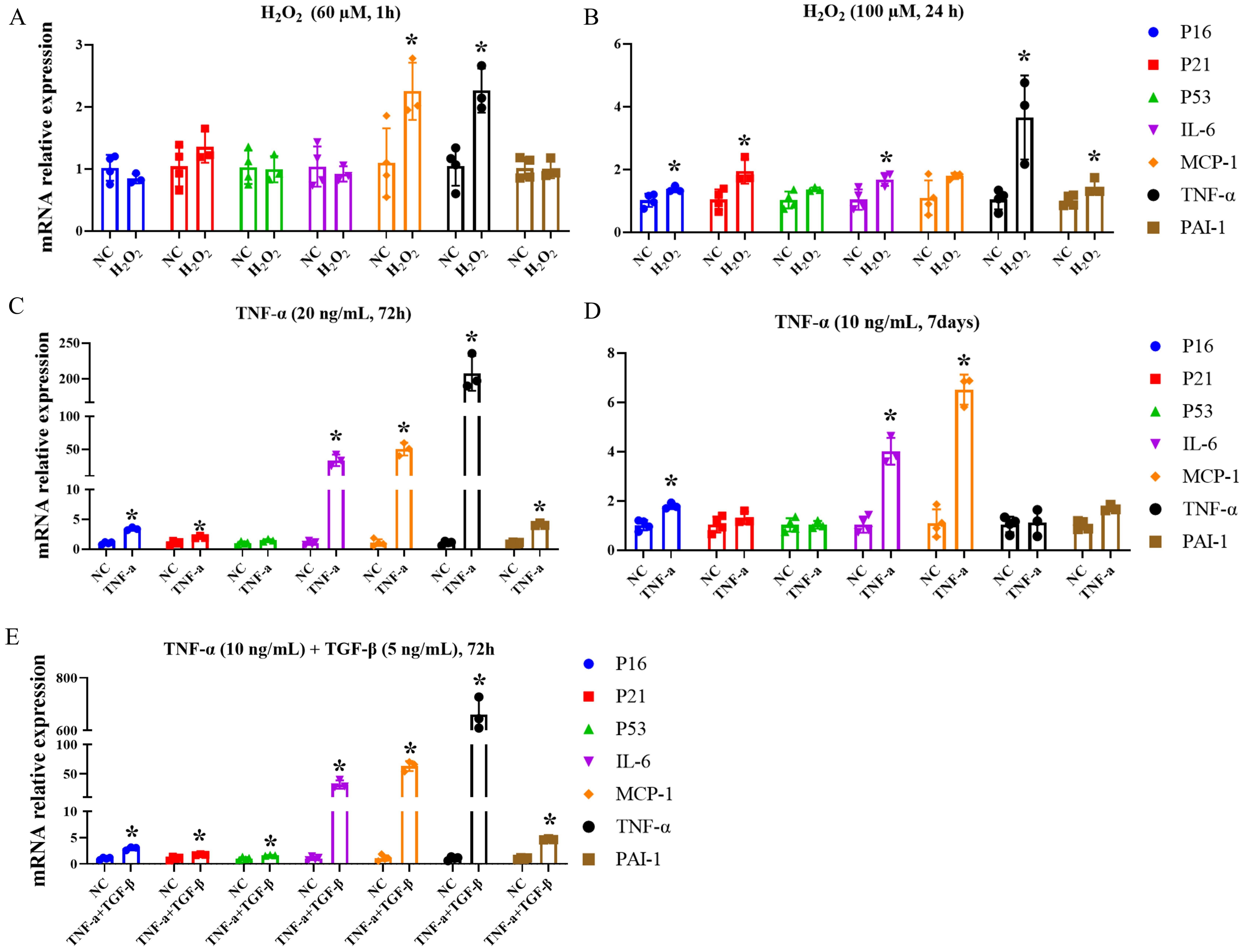

A
H2O2 (60 μM, 1h)
B
H2O2 (100 μM, 24 h)
C
TNF-α (20 ng/mL, 72h)
D
TNF-α (10 ng/mL, 7days)
E
TNF-α (10 ng/mL) + TGF-β (5 ng/mL), 72h
mRNA relative expression
NC
H2O2
TNF-a
TNF-a+TGF-β
P16
P21
P53
IL-6
MCP-1
TNF-α
PAI-1

Fig.S2

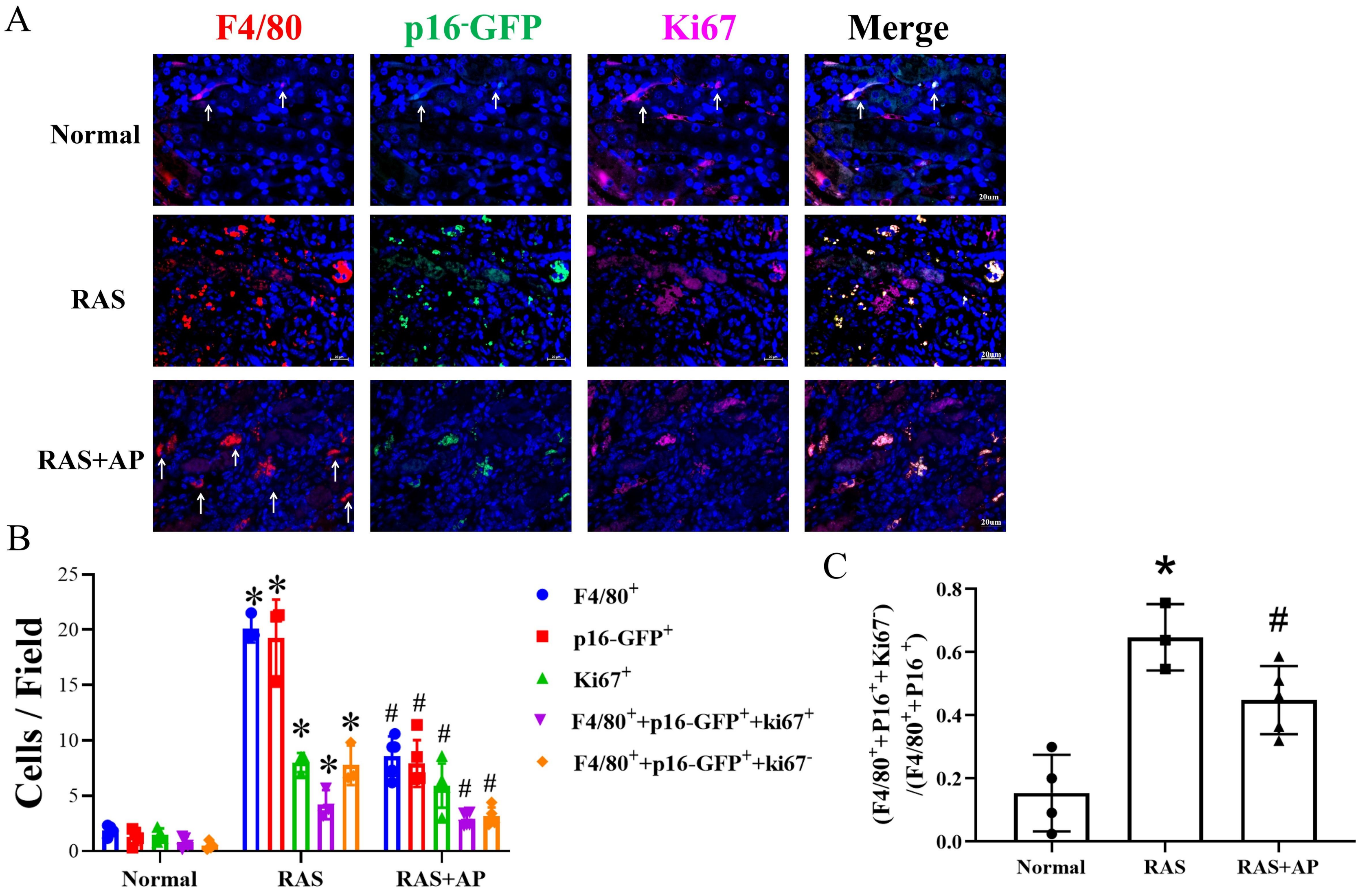
A
F4/80
p16-GFP
Ki67
Merge
Normal
RAS
RAS+AP
20um
B
Cells / Field
25
20
15
10
5
0
Normal
RAS
RAS+AP
F4/80+
p16-GFP+
Ki67+
F4/80+ +p16-GFP+ +ki67+
F4/80+ +p16-GFP+ +ki67-
C
(F4/80+ +P16+ +Ki67-) /(F4/80+ +P16+)
0.8
0.6
0.4
0.2
0.0
Normal
RAS
RAS+AP

Fig.S3

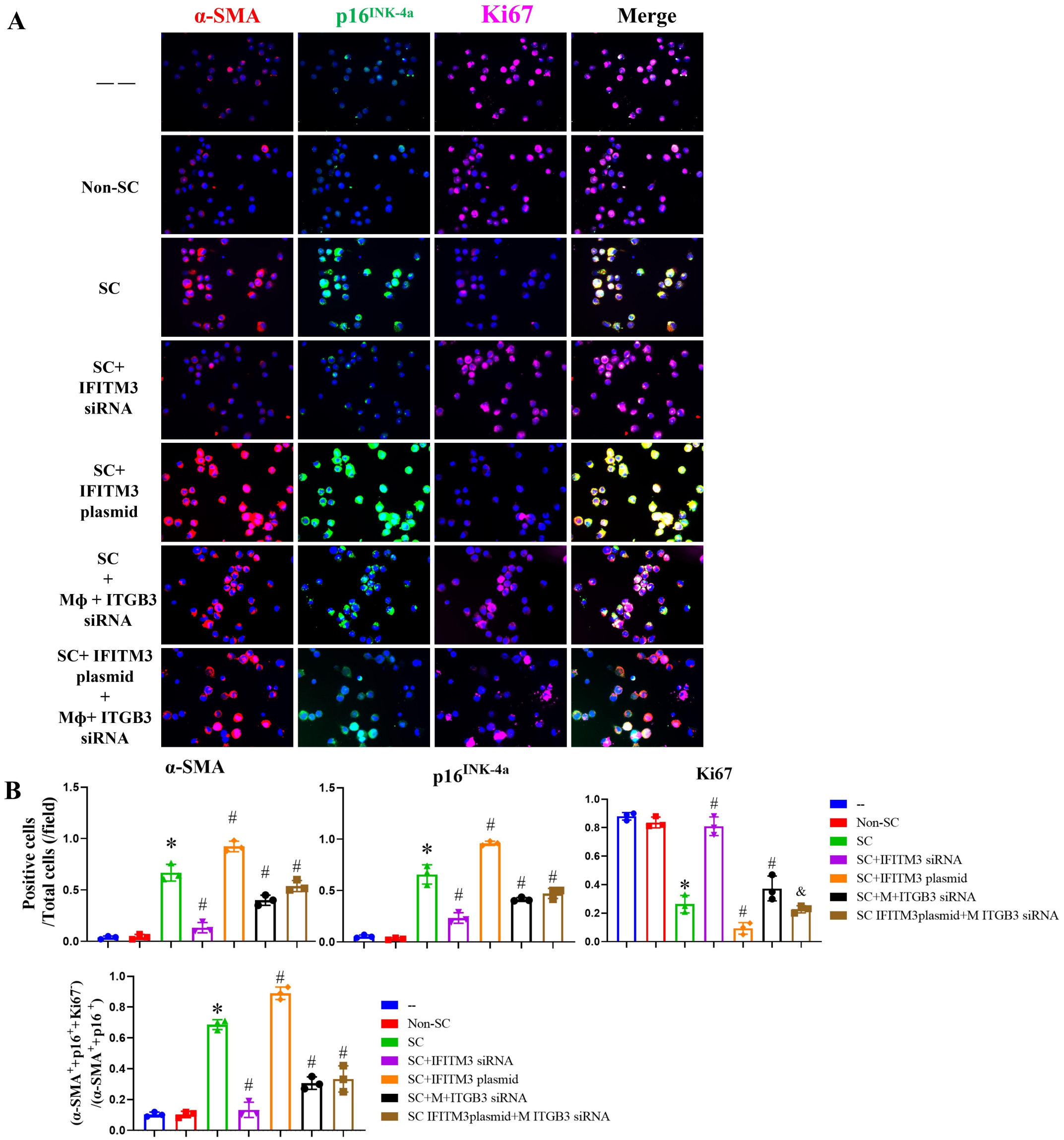
A
α-SMA
p16INK-4a
Ki67
Merge
——
Non-SC
SC
SC+ IFITM3 siRNA
SC+ IFITM3 plasmid
SC + Mϕ + ITGB3 siRNA
SC+ IFITM3 plasmid + Mϕ+ ITGB3 siRNA
B
α-SMA
p16INK-4a
Ki67
Positive cells /Total cells (/field)
(α-SMA+p16+Ki67-) /(α-SMA+p16+)
--
Non-SC
SC
SC+IFITM3 siRNA
SC+IFITM3 plasmid
SC+M+ITGB3 siRNA
SC IFITM3plasmid+M ITGB3 siRNA

Fig.S4

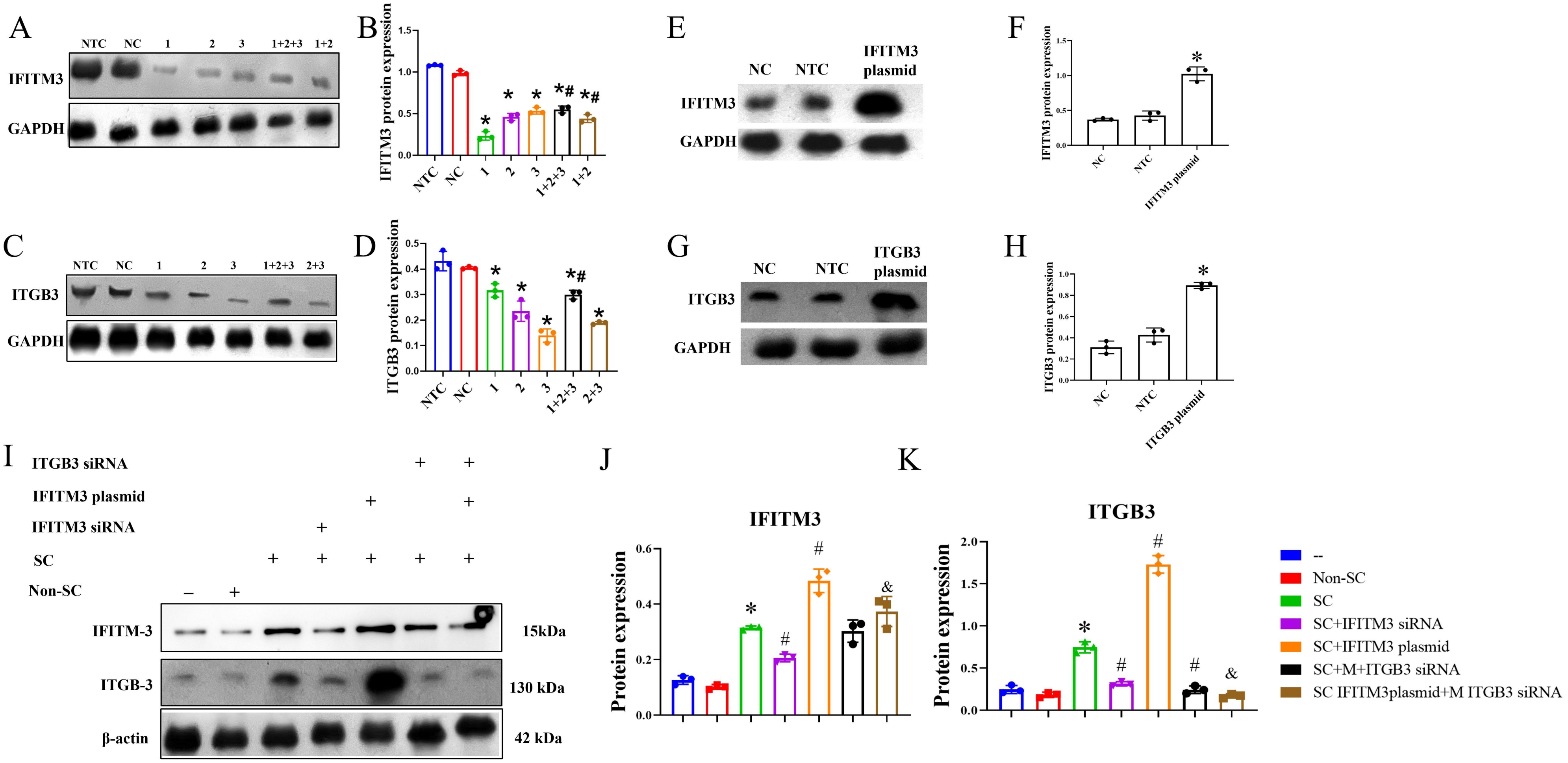
A
NTC NC 1 2 3 1+2+3 1+2
IFITM3
GAPDH
B
IFITM3 protein expression
NTC NC 1 2 3 1+2+3 1+2
C
NTC NC 1 2 3 1+2+3 2+3
ITGB3
GAPDH
D
ITGB3 protein expression
NTC NC 1 2 3 1+2+3 2+3
E
NC NTC IFITM3 plasmid
IFITM3
GAPDH
F
IFITM3 protein expression
NC NTC IFITM3 plasmid
G
NC NTC ITGB3 plasmid
ITGB3
GAPDH
H
ITGB3 protein expression
NC NTC ITGB3 plasmid
I
ITGB3 siRNA
IFITM3 plasmid
IFITM3 siRNA
SC
Non-SC
IFITM-3 15kDa
ITGB-3 130 kDa
β-actin 42 kDa
J
IFITM3
Protein expression
K
ITGB3
Protein expression
--
Non-SC
SC
SC+IFITM3 siRNA
SC+IFITM3 plasmid
SC+M+ITGB3 siRNA
SC IFITM3plasmid+M ITGB3 siRNA

Fig.S5

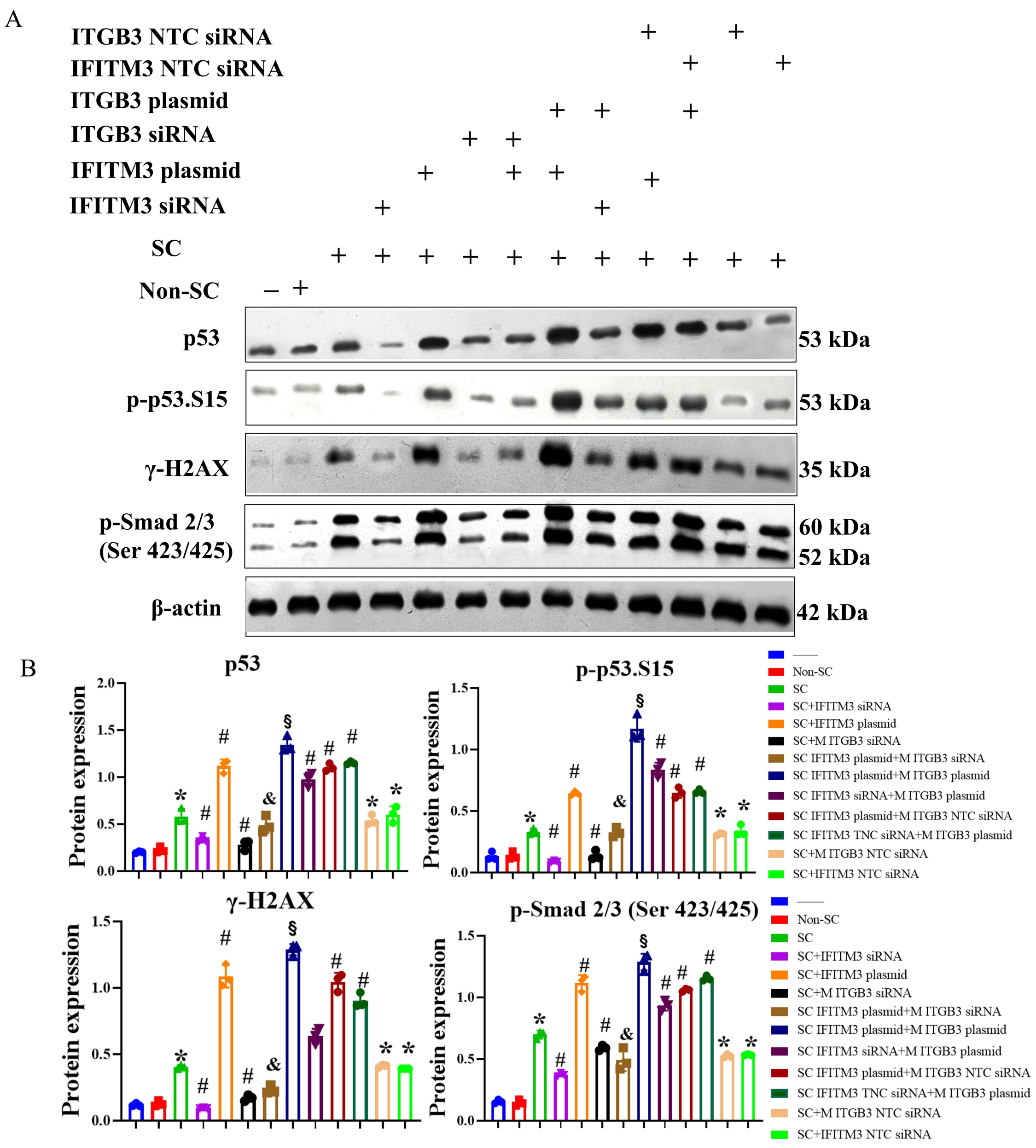
A
ITGB3 NTC siRNA
IFITM3 NTC siRNA
ITGB3 plasmid
ITGB3 siRNA
IFITM3 plasmid
IFITM3 siRNA
SC
Non-SC
p53
53 kDa
p-p53.S15
53 kDa
γ-H2AX
35 kDa
p-Smad 2/3
(Ser 423/425)
60 kDa
52 kDa
β-actin
42 kDa
B
p53
p-p53.S15
γ-H2AX
p-Smad 2/3 (Ser 423/425)
Protein expression
Non-SC
SC
SC+IFITM3 siRNA
SC+IFITM3 plasmid
SC+M ITGB3 siRNA
SC IFITM3 plasmid+M ITGB3 siRNA
SC IFITM3 plasmid+M ITGB3 plasmid
SC IFITM3 siRNA+M ITGB3 plasmid
SC IFITM3 plasmid+M ITGB3 NTC siRNA
SC IFITM3 TNC siRNA+M ITGB3 plasmid
SC+M ITGB3 NTC siRNA
SC+IFITM3 NTC siRNA

Fig.S6

Supplemental figure legends

Fig. S1 Macrophage clearance decreases MMT-mediated pro-fibrogenic activity.

(A) Triple-immunofluorescence images and (B) corresponding quantification revealed MMT cells co-expressing macrophage (F4/80, red), senescence activation (GFP-labeled p16, green), and myofibroblast (α-SMA, pink) markers in RAS kidneys compared to controls (Normal). The number of these MMT cells was significantly reduced in RAS kidneys treated with the macrophage-ablating reagent, clodronate (RAS+clodronate). Data are mean±SD (n=6 per group). *P<0.05 vs. Normal; #P<0.05 vs. RAS. Scale bar: 20μm (A). p16-GFP: $p16^{INK\text{-}4a+}$ labeled with green fluorescent protein (GFP).

Fig. S2 Comparison of approaches to induce senescence in HRPTEpiC.

We compared various approaches to induce senescence in HRPTEpiC, including H_2O_2 (A, 60μM, 1h; B, 100μM, 24h), TNF-α (C, 20 ng/mL, 72h; D, 10 ng/mL, 7 days), and TNF-α (10 ng/mL)+TGF-β (5 ng/mL), 3 days (E). Our PCR data demonstrated that the combination of TNF-α (10 ng/mL) and TGF-β (5 ng/mL) for 3 days was the most effective and consistent in upregulating gene expression of all the senescence markers. Data are mean±SD (n=3/group). *P<0.05 vs. Normal.

Fig. S3 $p16^{INK\text{-}4a+}$ macrophages show decreased proliferation ability *in vivo*

Triple-immunofluorescence analysis identified $p16^{INK\text{-}4a+}$ macrophages co-expressing macrophage (F4/80, red), senescence activation (p16-GFP, green), and proliferation (Ki67, pink) markers in RAS kidneys compared to controls (Normal). The number of these cells was significantly reduced in RAS kidneys treated with the $p16^{INK\text{-}4a+}$ cell apoptosis inducer, AP20187 (RAS+AP20187). Data are mean±SD (n=6/group). *P<0.05 vs. Normal; #P<0.05 vs. RAS. Scale bar: 20μm (A). p16-GFP: $p16^{INK\text{-}4a+}$ be labeled by green fluorescent protein (GFP).

Fig. S4 MMT macrophages show decreased proliferation ability *in vitro*

To assess the development of cell cycle arrest (which characterized cellular senescence) in

MMT macrophages, we performed triple immunofluorescence staining to identify cells co-expressing myofibroblast (α-SMA, red), senescence activation (*p16*$^{INK\text{-}4a}$, green), and proliferation (Ki67, pink) markers. This approach demonstrated that macrophages undergoing MMT *in vitro*, in the presence of senescent cells, exhibited reduced proliferative capacity, consistent with the acquisition of a senescent phenotype. Scale bar: 20μm (A). Data are mean ± SD (n=3/group). *P<0.05 vs. Normal control (NC) and Non-SC groups; #P<0.05 vs. SC group; and &P<0.05 vs. SC+IFITM3 over-expressing groups.

Fig. S5 Confirmation of the effects of IFITM3 (in HRPTEpiC) and ITGB3 (in Macrophages) siRNA and plasmid on IFITM3 and ITGB3 protein expression.

(A-B): IFITM3 expression in HRPTEpiC transfected with three distinct IFITM3 siRNAs or a non-target control (NTC) siRNA. Among the siRNAs tested, IFITM3 siRNA-1 most effectively downregulated IFITM3 protein levels. Data are mean±SD (n=3/group). *p<0.05 vs NC group, $^{\#}$p<0.05 vs IFITM3 siRNA-1 group. (C-D): ITGB3 protein levels in macrophages transfected with three distinct ITGB3 siRNAs or an NTC siRNA. Among the siRNAs tested, ITGB3 siRNA-3 most effectively reduced ITGB3 protein expression. Data are mean±SD (n=3/group). *p<0.05 vs NC, $^{\#}$p<0.05 vs ITGB3 siRNA-3. (E-F): Transfection with the IFITM3 plasmid successfully upregulated IFITM3 protein levels in HRPTEpiC. (G-H): Similarly, transfection with the ITGB3 plasmid increased ITGB3 protein levels in macrophages. Data are mean±SD (n=3/group). *P<0.05 vs. NC. HRPTEpiC: Human renal proximal tubular epithelial cells, NC: Normal Control; siRNA: small interfering RNA. (I-K): IFITM3 and ITGB3 were manipulated in senescent human renal proximal tubular epithelial cells (SC) and macrophages, respectively. Macrophages were subsequently collected for Western blot analysis to assess proteins expression of IFITM3 and ITGB3. Knockdown of ITGB3 or IFITM3 using siRNA individually reduced the expression of IFITM3 and ITGB3. However, these inhibitory effects

were attenuated when IFITM3 was overexpressed in HRPTEpiC. Data are mean±SD (n=3/group). *P<0.05 vs. Normal control (NC) and Non-SC groups; #P<0.05 vs. SC group; and &P<0.05 vs. SC+IFITM3 over-expressing groups.

Fig. S6 The effects of IFITM-3 and ITGB-3 overexpression and silencing on senescence and p-Smad 2/3 in macrophages.

Overexpression and silencing of IFITM3 and ITGB3 were manipulated in senescent (SC) human renal proximal tubular epithelial cells (HRPTEpiC) and macrophages, respectively. Macrophages were then collected for Western blot analysis of senescence markers (p53, p-p53 (Ser15), and γ-H2AX) and p-Smad2/3 (Ser423/425). Non-targeting (NTC) siRNA was used as a control to exclude off-target effects of siRNA transfection. The expression of the senescence markers and p-Smad2/3 (Ser423/425) proteins in macrophages increased after co-culture with senescent HRPTEpiC. These increases were significantly reduced following treatment with IFITM3-siRNA or ITGB3-siRNA. However, the blunting effects of siRNA treatment were attenuated when IFITM3 and ITGB3 were overexpressed, highlighting their roles in sustaining senescence and pathway activation in macrophages. Data are mean±SD (n=3/group). *P<0.05 vs. Normal control (NC) and Non-SC groups; #P<0.05 vs. SC group; &P<0.05 vs. SC+IFITM3 over-expressing groups, and § P<0.05 vs. SC+ IFITM3 plasmid+Mϕ+ ITGB3 siRNA.
