# Supplementary material for: Cellular senescence promotes macrophage-to-myofibroblast transition in chronic ischemic renal disease
Source: Cell Death Dis. 2025 May 10;16(1):372. doi: 10.1038/s41419-025-07666-1 (PMC12065848; doi:10.1038/s41419-025-07666-1)

Collagen smad

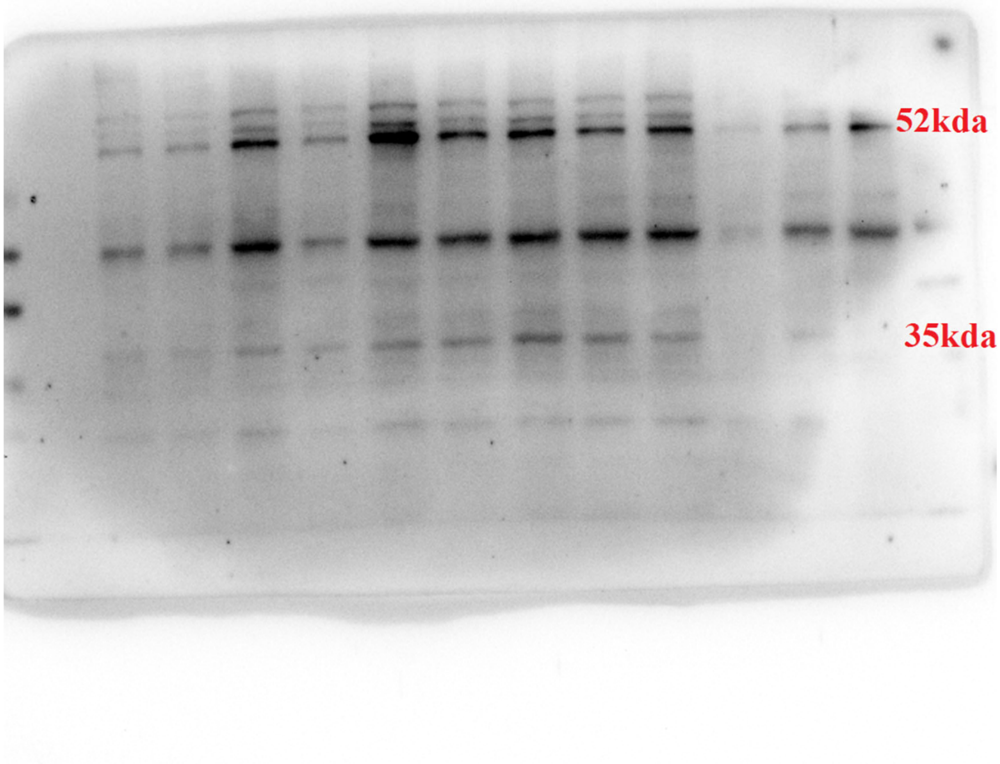

Ifitm3 plasmid

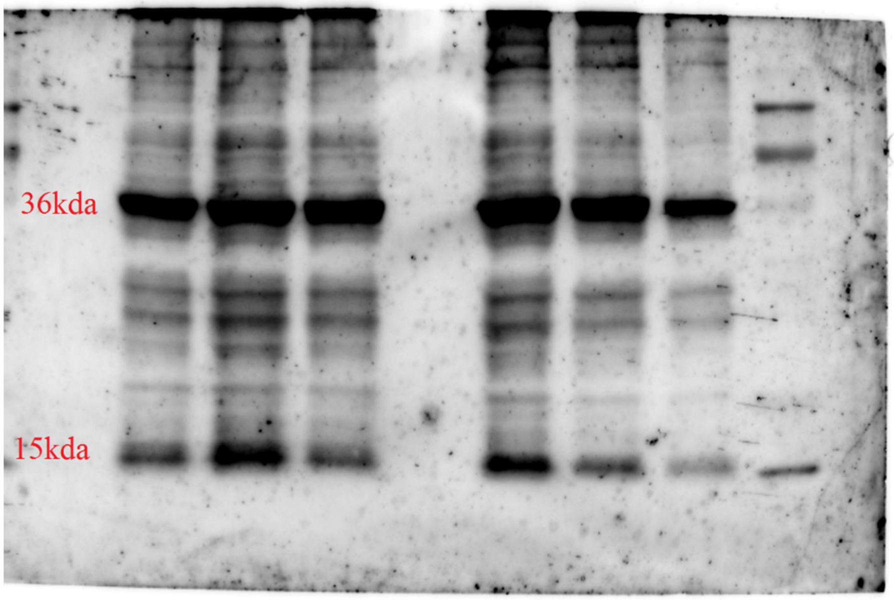

Ifitm3 siRNA

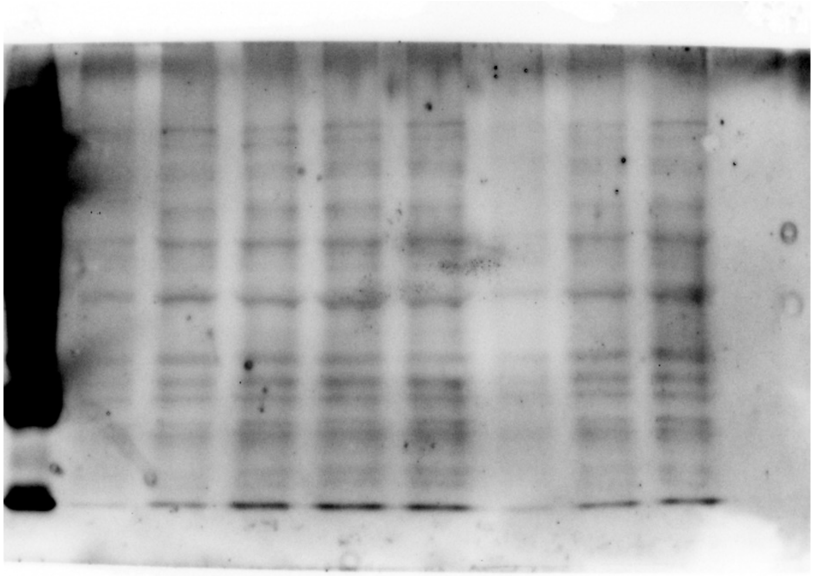

Ifitm3

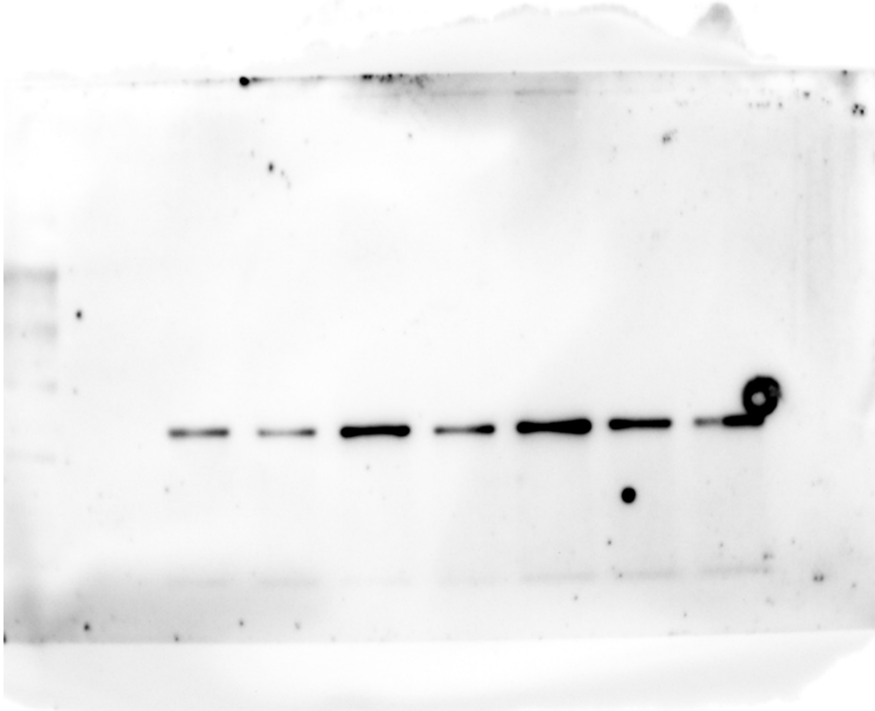

ITGB3 siRNA

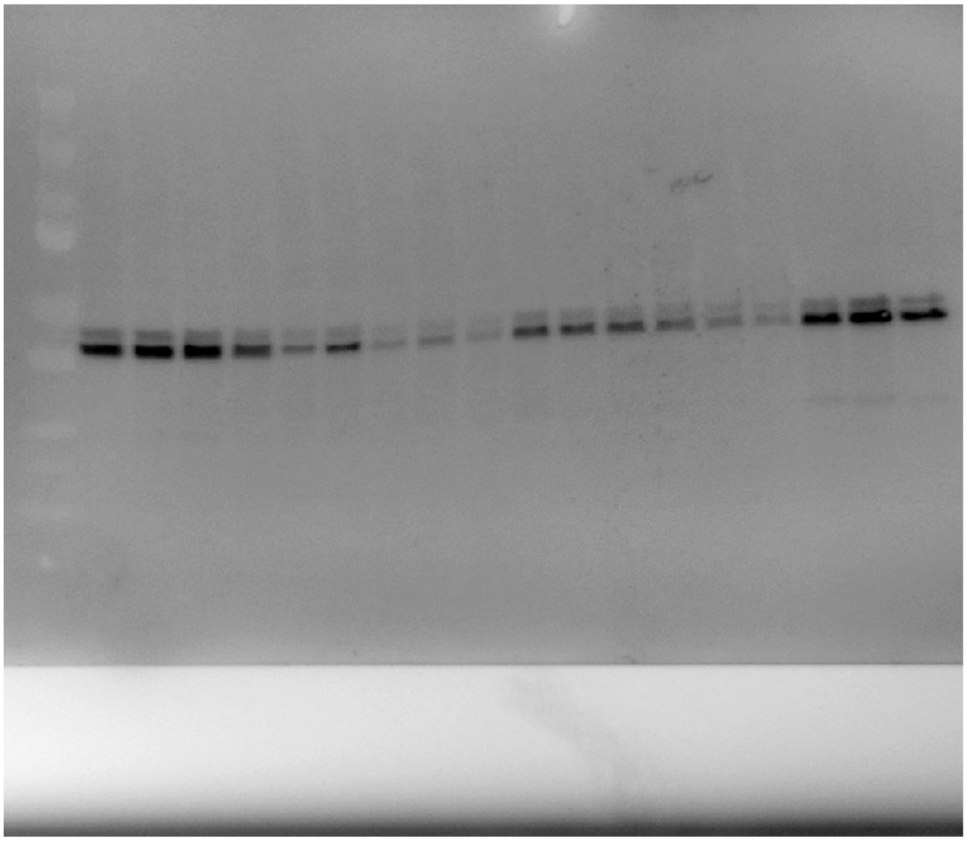

ITGB3

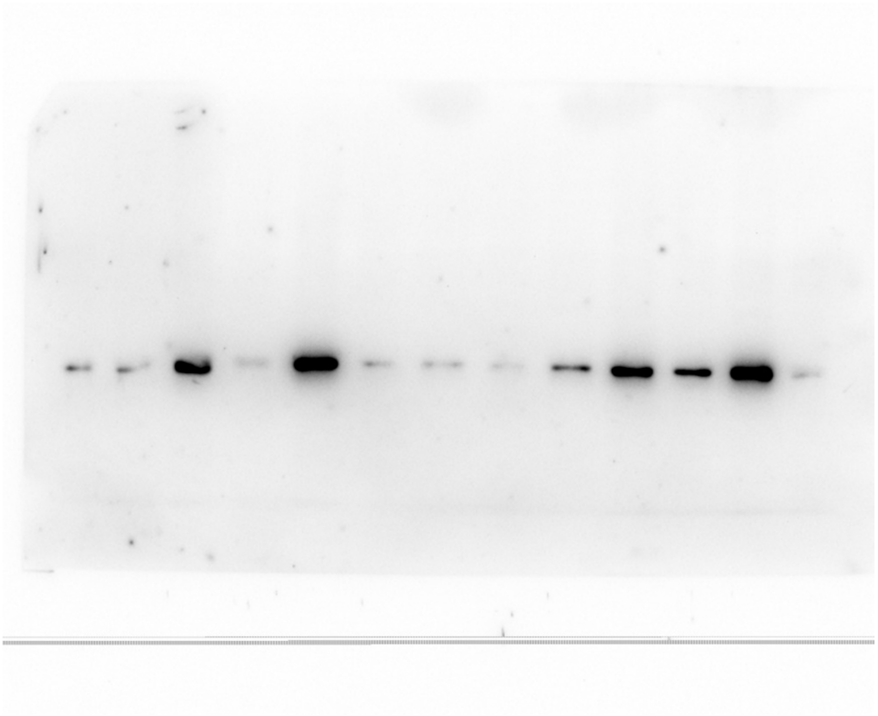

Normal

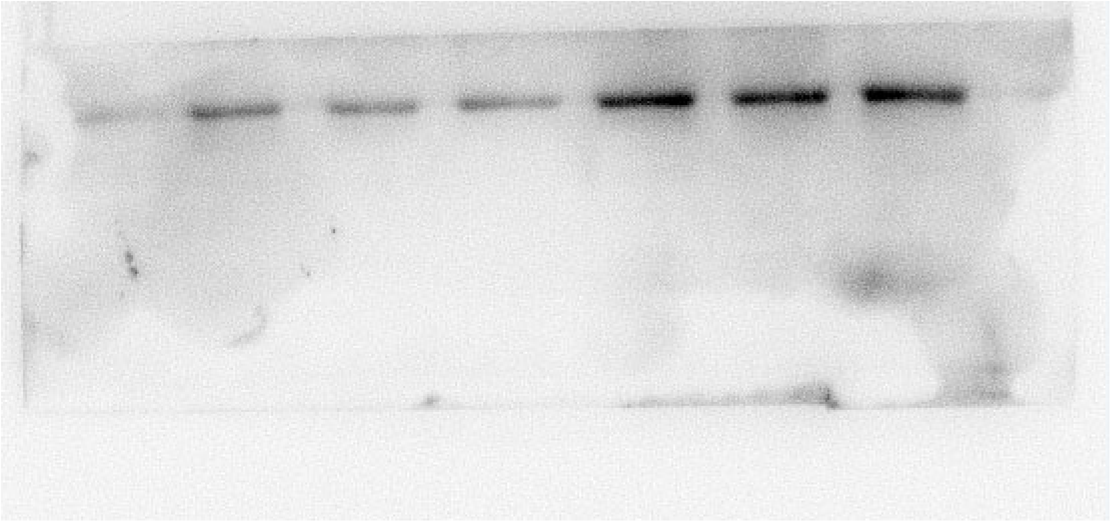

P21

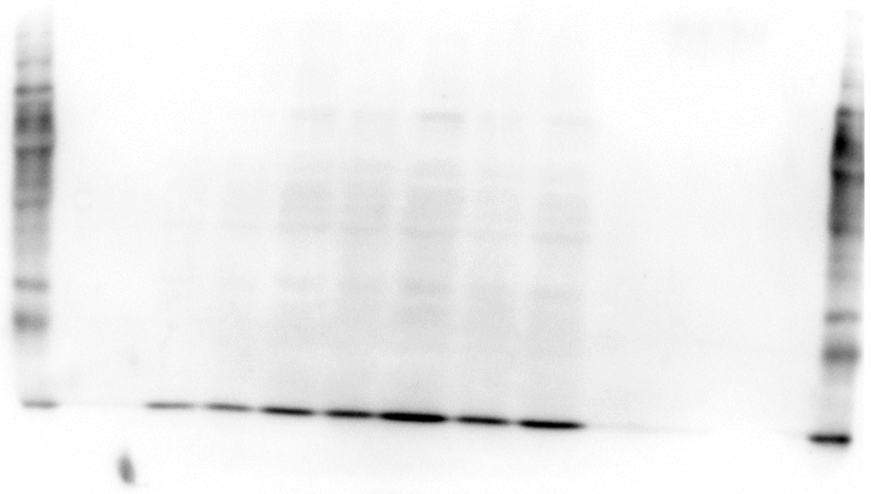

Tgfb

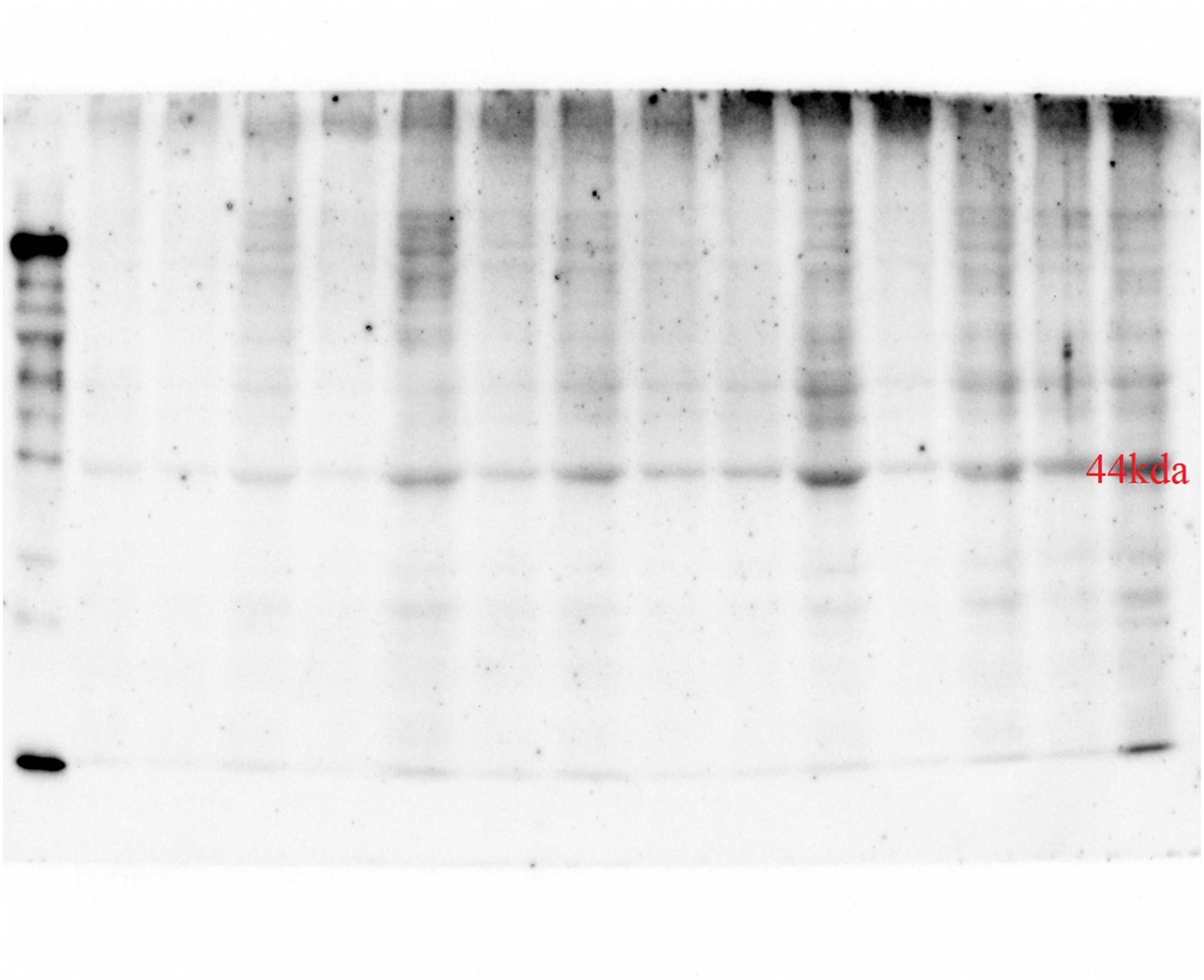

Fig 3I IFITM3

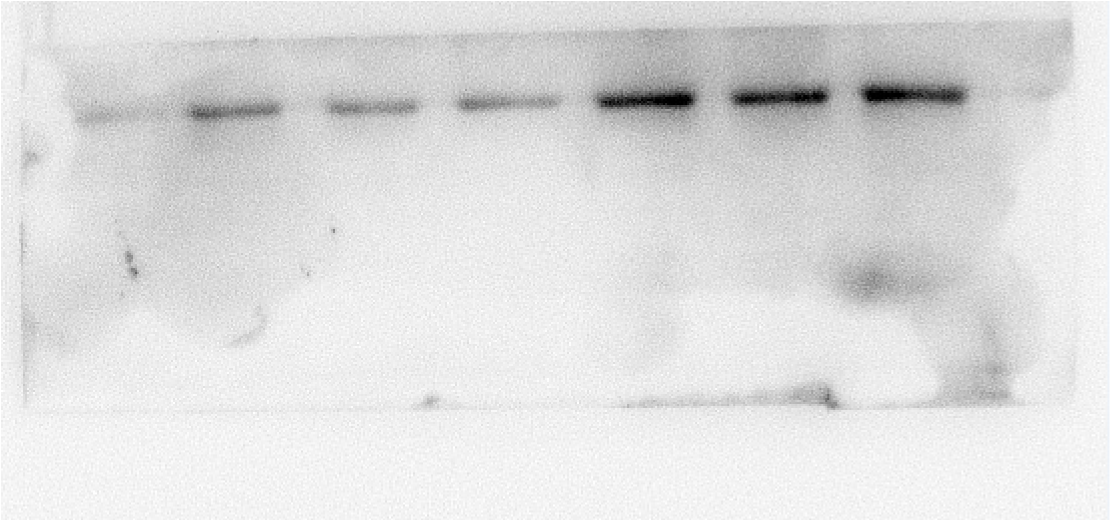

Fig S5-1 p21

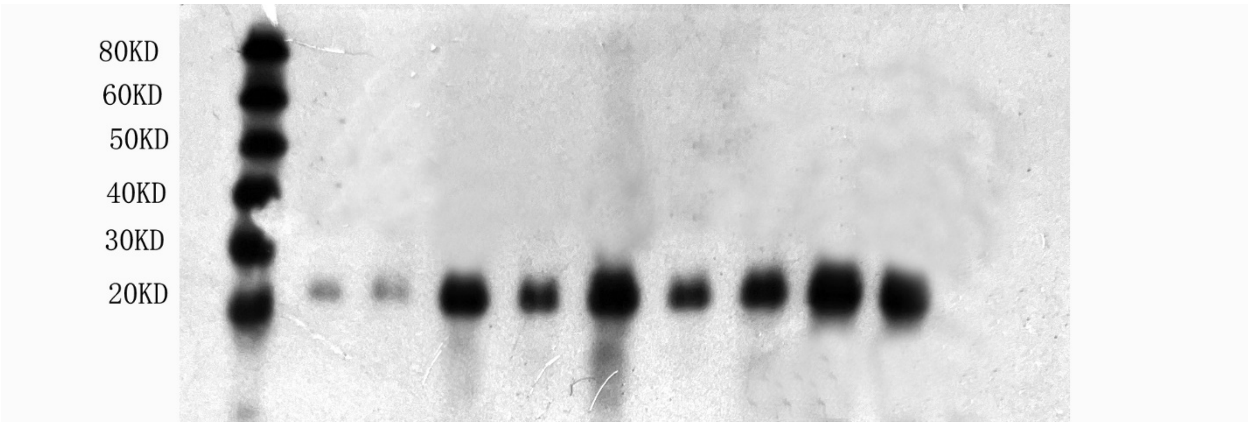

Fig S6-1 p53

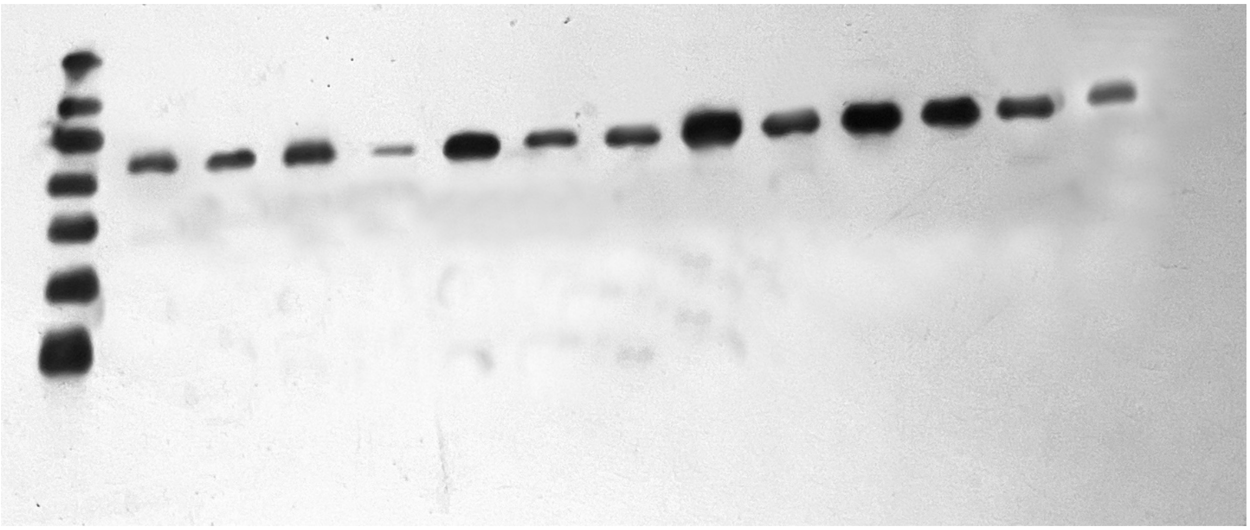

Fig S6-2 p-p53

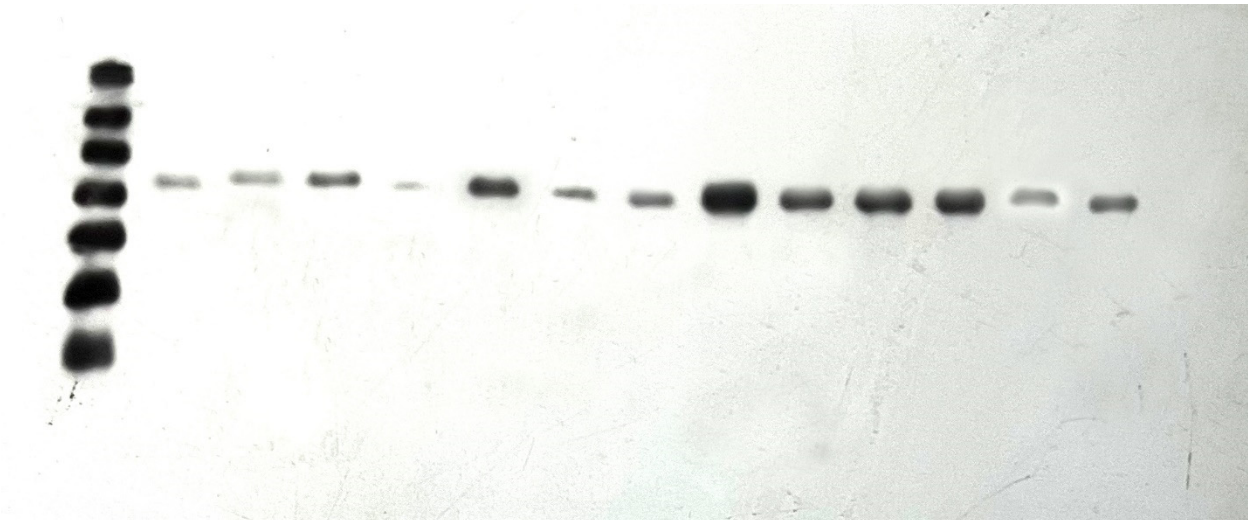

Fig S6-4 γ-H2AX

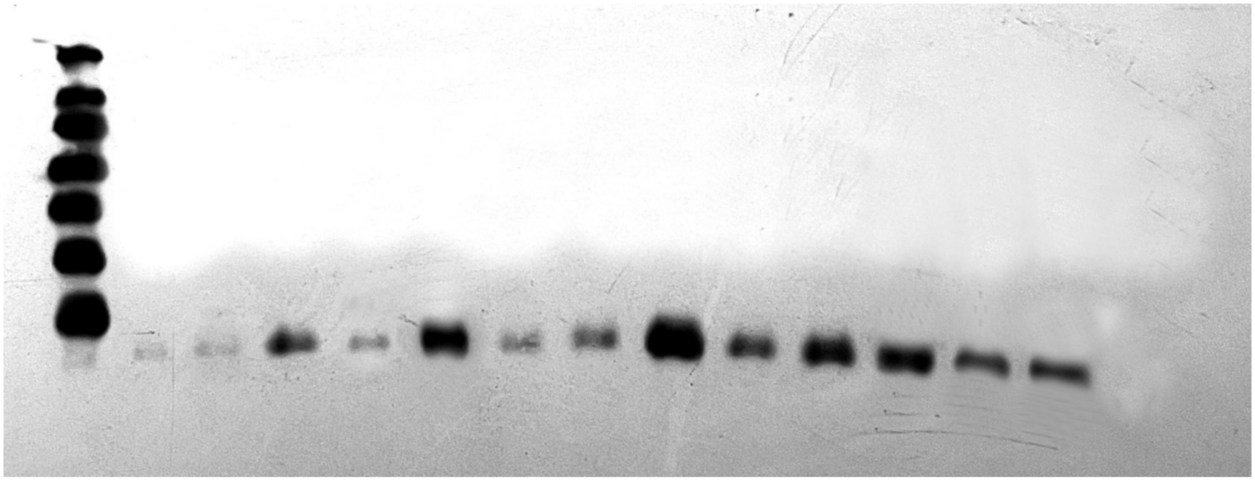

Fig6-1 collagen I

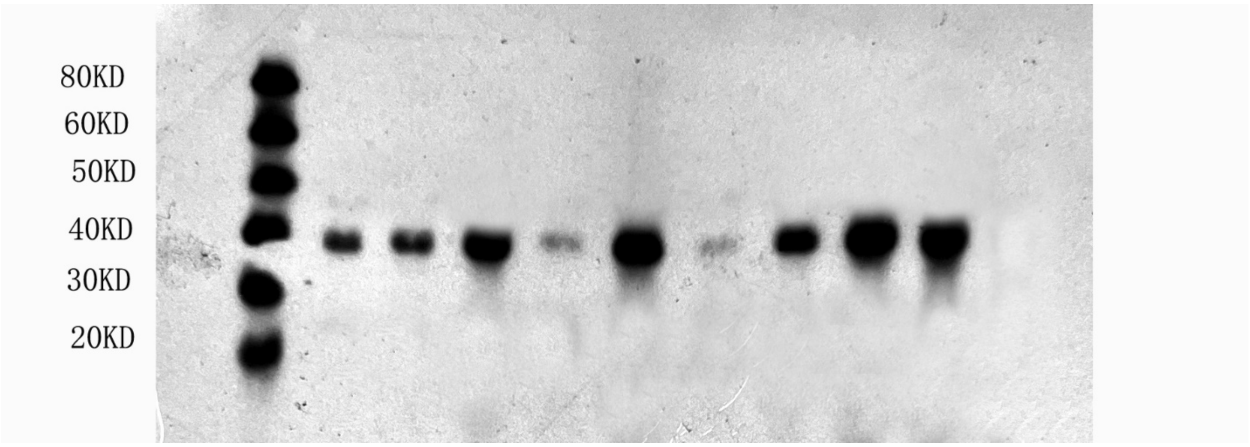

Fig6-2 TGF beta

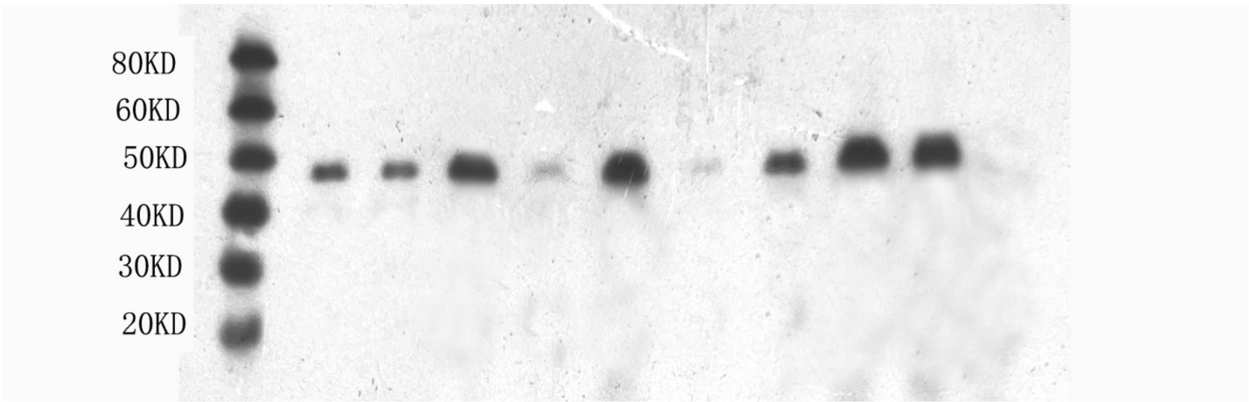

Fig6-3 p-sma2/3

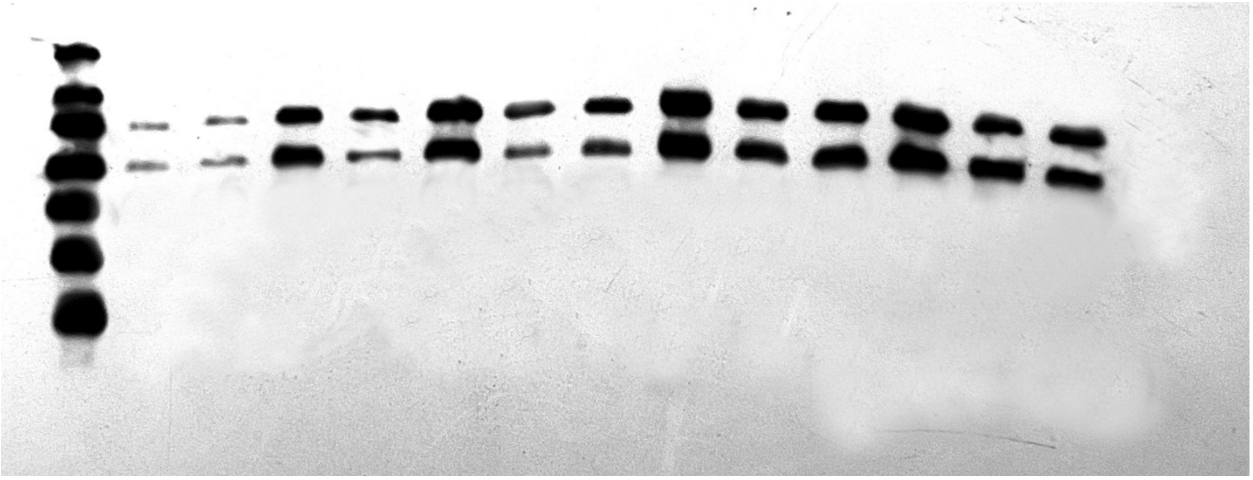

Fig6-4 beta actin 1

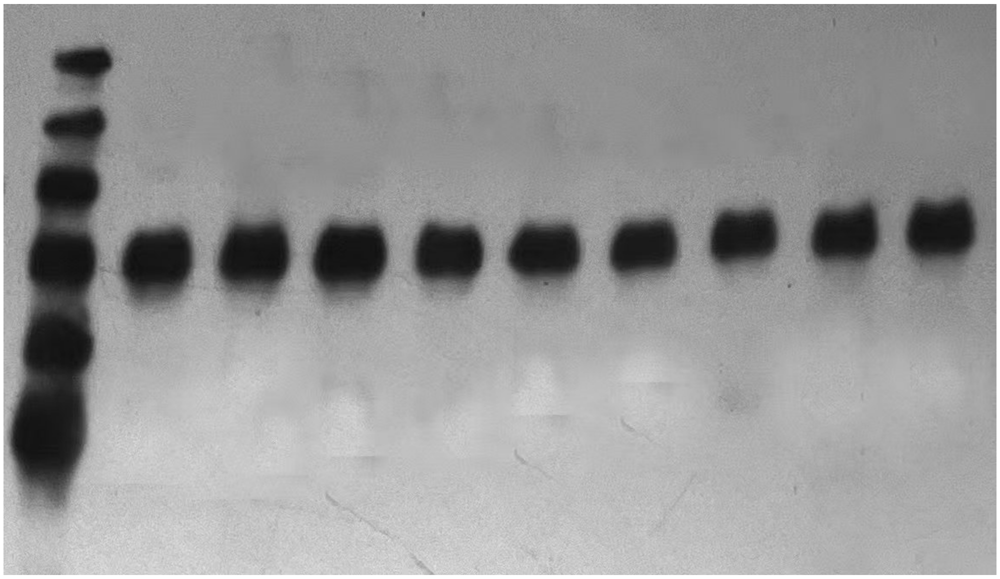

Fig S5-1 GAPDH

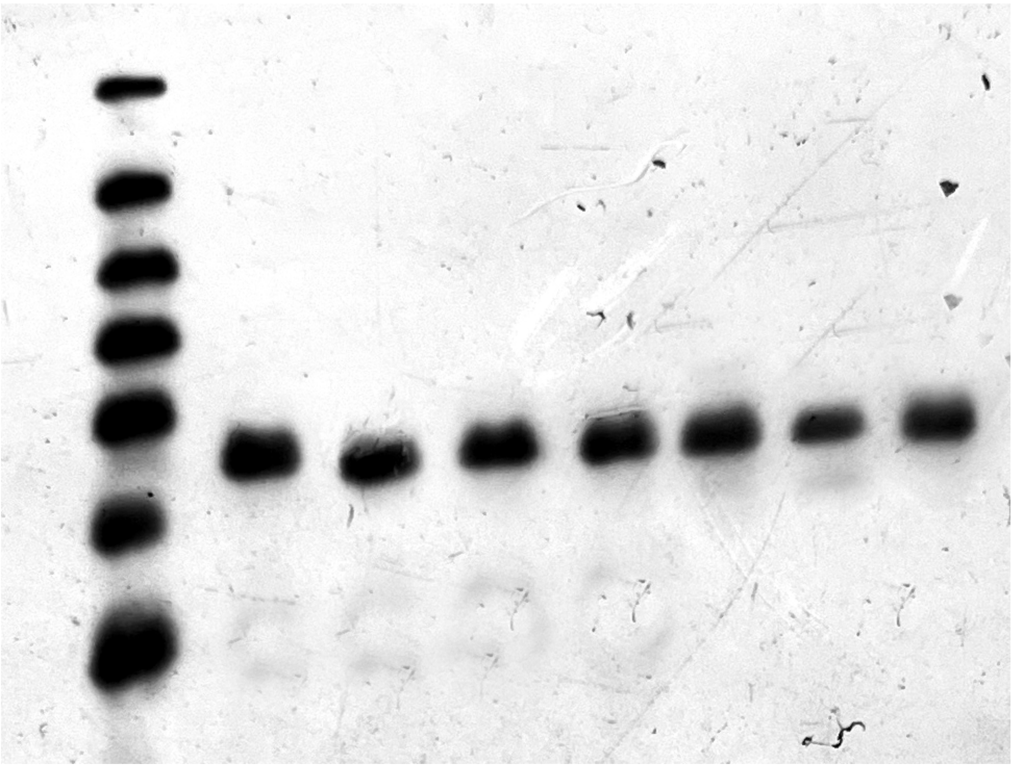

Fig S5-1 IFITM3

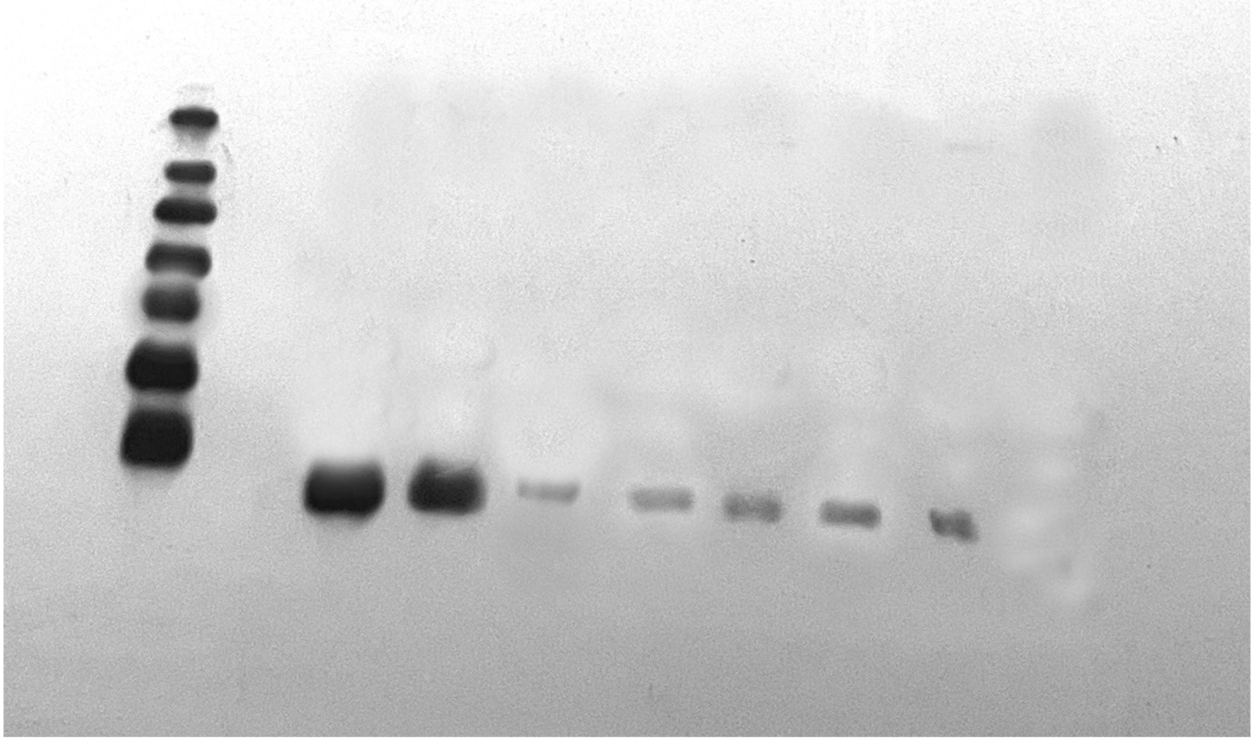

Fig S5-2 GAPDH-2

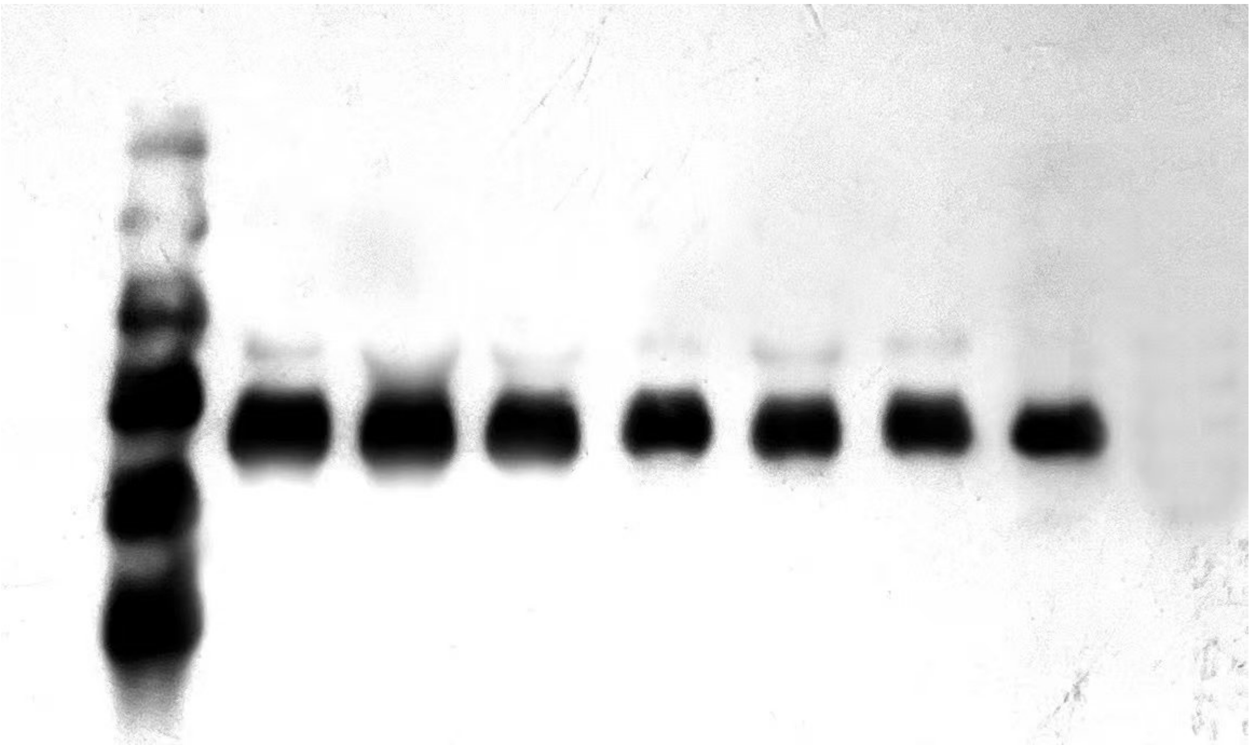

Fig S5-2 ITGB3

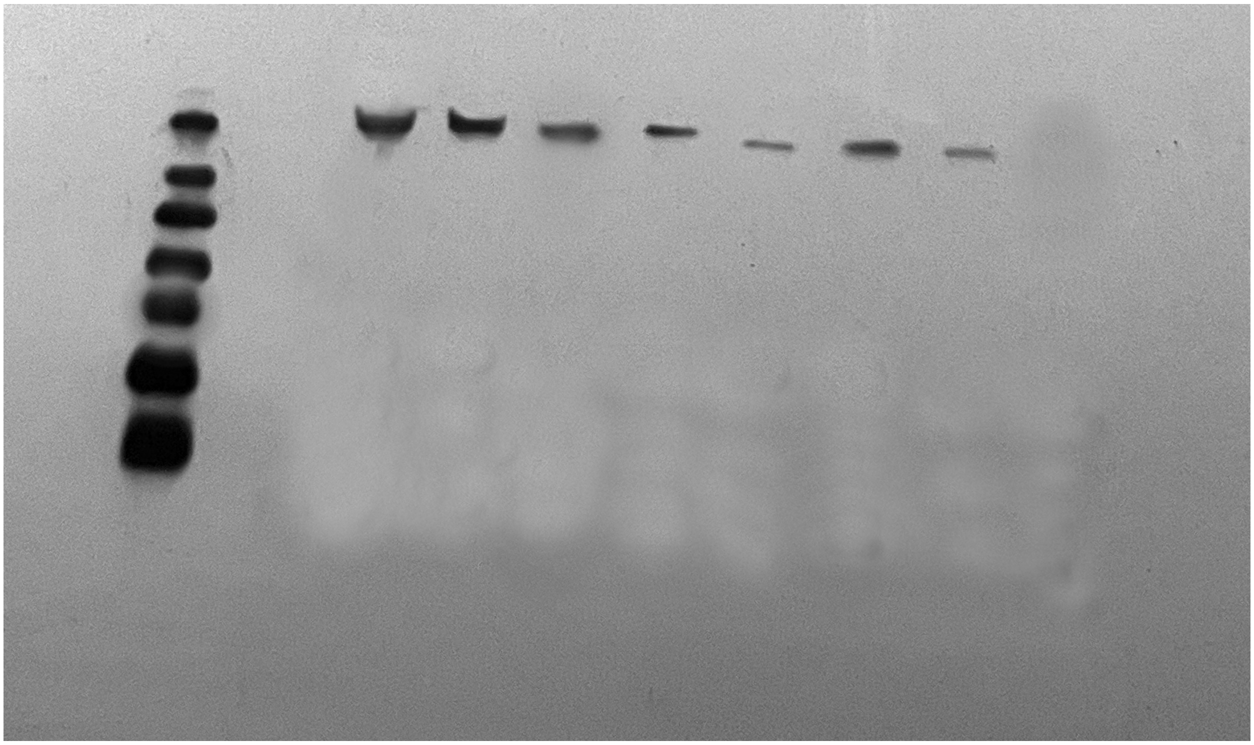

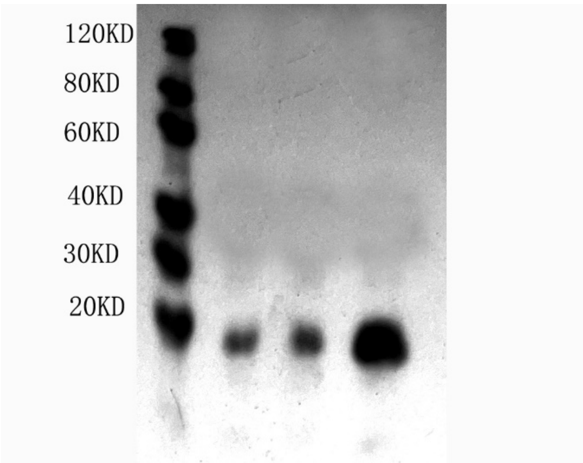

Fig S5-3 IFITM3

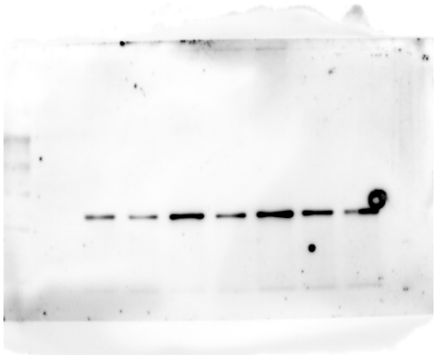

Fig S5-4 ITGB3

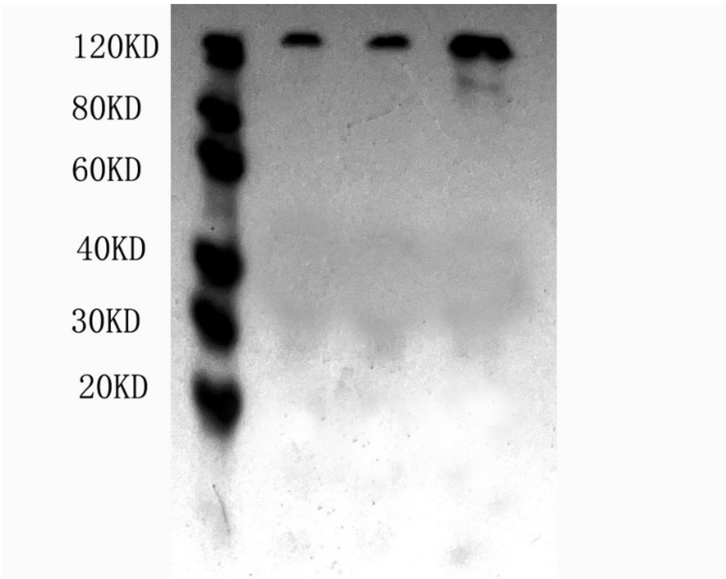

Fig S5-4 ITGB3-1

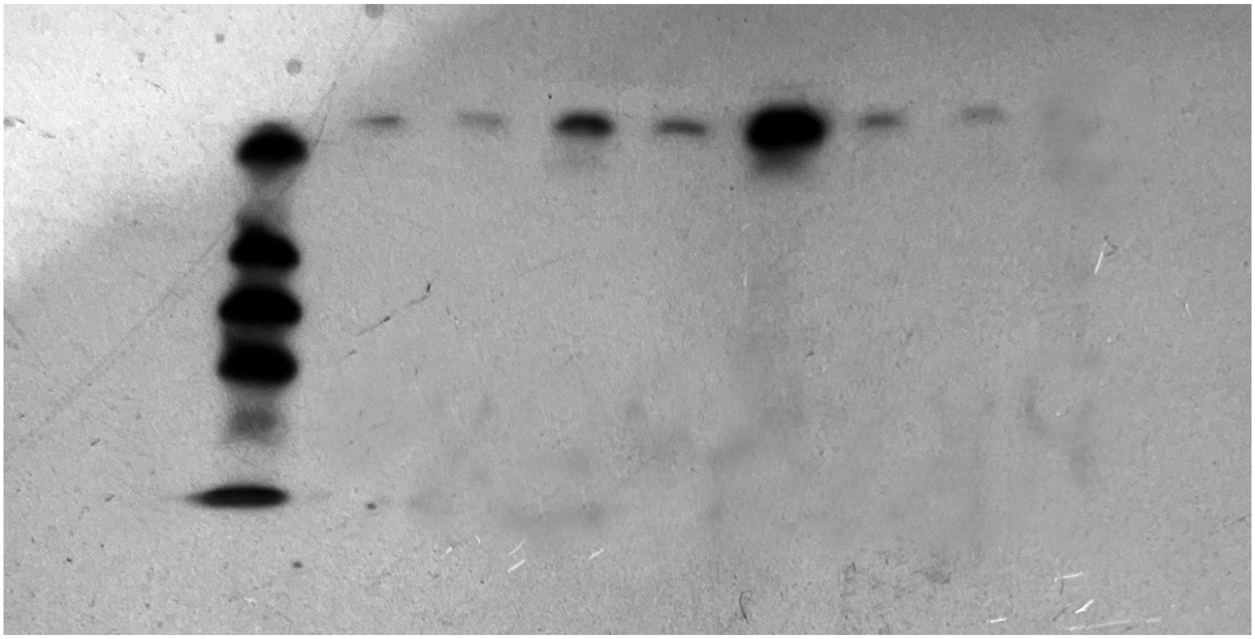

Supplement: Supplementary file 3 — Western Blot Gels [file 41419_2025_7666_MOESM3_ESM.pdf]
